# Schlemm's Canal Is a Unique Vessel with a Combination of Blood Vascular and Lymphatic Phenotypes that Forms by a Novel Developmental Process

**DOI:** 10.1371/journal.pbio.1001912

**Published:** 2014-07-22

**Authors:** Krishnakumar Kizhatil, Margaret Ryan, Jeffrey K. Marchant, Stephen Henrich, Simon W. M. John

**Affiliations:** 1 The Howard Hughes Medical Institute, and The Jackson Laboratory, Bar Harbor, Maine, United States of America; 2 Department of Integrative Physiology and Pathobiology, Tufts University School of Medicine, Boston, Massachusetts, United States of America; 3 Department of Ophthalmology and Sackler School of Graduate Biomedical Sciences, Tufts University School of Medicine, Boston, Massachusetts, United States of America; Duke University Medical Center, United States of America

## Abstract

Schlemm's canal (SC) plays central roles in ocular physiology. These roles depend on the molecular phenotypes of SC endothelial cells (SECs). Both the specific phenotype of SECs and development of SC remain poorly defined. To allow a modern and extensive analysis of SC and its origins, we developed a new whole-mount procedure to visualize its development in the context of surrounding tissues. We then applied genetic lineage tracing, specific-fluorescent reporter genes, immunofluorescence, high-resolution confocal microscopy, and three-dimensional (3D) rendering to study SC. Using these techniques, we show that SECs have a unique phenotype that is a blend of both blood and lymphatic endothelial cell phenotypes. By analyzing whole mounts of postnatal mouse eyes progressively to adulthood, we show that SC develops from blood vessels through a newly discovered process that we name “canalogenesis.” Functional inhibition of KDR (VEGFR2), a critical receptor in initiating angiogenesis, shows that this receptor is required during canalogenesis. Unlike angiogenesis and similar to stages of vasculogenesis, during canalogenesis tip cells divide and form branched chains prior to vessel formation. Differing from both angiogenesis and vasculogenesis, during canalogenesis SECs express *Prox1*, a master regulator of lymphangiogenesis and lymphatic phenotypes. Thus, SC development resembles a blend of vascular developmental programs. These advances define SC as a unique vessel with a combination of blood vascular and lymphatic phenotypes. They are important for dissecting its functions that are essential for ocular health and normal vision.

## Abstract 2

A draining vessel in the eye arises via a novel hybrid process of vascular development and is important for understanding ocular fluid homeostasis and glaucoma.

## Author Summary

Schlemm's canal serves as a drainage tube for fluid from the anterior chamber of the eye and is directly relevant to glaucoma, a disease that causes vision loss in over 70 million people. Aqueous humor enters the canal and then drains into connected veins. Molecular understanding of the development of Schlemm's canal and its drainage functions has remained limited. We provide a detailed characterization of Schlemm's canal development, and in so doing discover a novel process of vascular development that we name “canalogenesis.” We show that although the process requires a functional KDR receptor, which is also critical in blood vessel development, the endothelial cells of Schlemm's canal have a unique hybrid molecular phenotype, expressing proteins that are characteristic of both blood and lymphatic vessels. Of note, the expression of *Prox1*, a master regulator of lymphatic fate, and other lymphatic proteins are largely restricted to specialized cells of the inner wall of Schlemm's canal through which the aqueous humor passes as it exits the eye. Thus, *Prox1* and other lymphatic proteins may be critical for the functional specialization of these cells for aqueous humor drainage. Schlemm's canal is thus a unique vessel with a combination of blood vascular and lymphatic characteristics.

## Introduction

Although Schlemm's canal (SC) has central roles in ocular physiology and homeostasis, its development, mature phenotype, and molecular processes are poorly understood [1]–[3]. SC has a critical role in aqueous humor drainage (AQH) from the eye, a process that regulates the intraocular pressure (IOP) [1],[2],[4],[5]. Abnormal resistance to AQH drainage results in IOP elevation, a key factor contributing to glaucoma [2]. Glaucoma is one of the most common neurodegenerative diseases and will affect an estimated 80 million people by the end of this decade [6]. SC is also important for anterior chamber associated immune deviation (ACAID), a form of immune tolerance [3]. During ACAID, immune cells are exposed to an antigen in the eye and then exit the eye via SC. From SC they return to the systemic circulation via blood vessels to which SC is connected [7],[8]. After exiting SC, these cells induce a systemic suppression of immune responses to that antigen. Thus, SC is a unique and important vessel that needs to be better understood.

SC is a flattened tube made of endothelial cells, which encircles the anterior portion of the eye. It is embedded within the ocular wall in the region connecting the cornea and sclera that is known as the limbus. Specifically, SC is located in tissue of the iridiocorneal angle (angle formed by the iris and cornea) [2],[9]. The inner wall of SC consists of morphologically specialized endothelial cells and their basement membrane, which provide a final barrier to the drainage (outflow) of AQH and the exit of immune cells from the eye [1],[2],[7],[8]. SC endothelial cells (SECs) and their specialized basement membrane are likely to contribute a key source of resistance to AQH outflow. As immune cell behavior is modulated by interactions with endothelial cells, SECs are likely to have important molecular roles in immune tolerance. However, many mechanistic questions about the functions of SC remain unanswered.

Determining the origin and phenotype of the SC and its endothelial cells is key to understanding its roles in ocular homeostasis and immune regulation. Based on a variety of features including marker expression, nature of cellular junctions, direction of fluid flow, and cellular morphology, SECs have similarities and differences to both blood endothelial cells (BECs) and lymphatic endothelial cells (LECs) and may be a unique endothelial cell type [5]. However, studies investigating the expression of lymphatic markers detected none in both human and mouse SC [10]–[12]. Thus, the molecular nature of SECs remains controversial.

SC is proposed to develop from blood vasculature, but further investigation of its tissue origins is required as existing models of SC development differ significantly. In the first model, SC forms from a blood filled venous plexus anterior to the trabecular anlage (the anlage that gives rise to the trabecular meshwork, which is adjacent to SC in mature eyes) [13]–[17]. In the second model, SC forms from blood vessels originating from a more superficial limbal plexus [18]. Our previous studies suggested that SC forms by the penetration of existing vessels to a location adjacent to the trabecular anlage and that they anastomose to make SC [19]. These previous studies are limited by the use of techniques that sample small regions of tissue in two-dimensional sections (using light and electron microscopy). They provide no molecular detail about mechanisms and have not considered or tested a lymphatic origin for SC.

To allow a modern, more detailed and extensive analysis of the SC phenotype and its developmental origins, we developed a new limbal whole-mount procedure and applied lineage-specific fluorescent reporter genes, high-resolution confocal microscopy, and three-dimensional (3D) rendering to study large regions of the developing limbus. We show that in addition to expressing markers of BECs, developing and mature SECs express PROX1. PROX1 (prospero-related homeobox1) is well established to be an important regulatory protein, which is necessary and sufficient for acquiring a lymphatic fate [20]. Furthermore, we discover that SC develops by a previously unknown process, which has commonalities and differences to the three described processes of vascular development—vasculogenesis, angiogenesis, and lymphangiogenesis.

## Results

### Developing and Adult SC Can Be Visualized in 3D Whole Mounts

**Figure 1:**
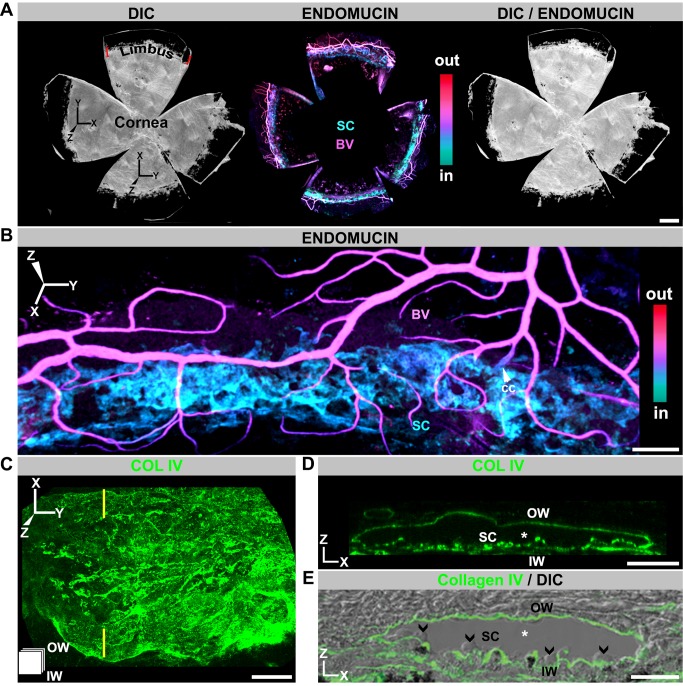
SC visualized in 3D using whole mounts. (A) Enface view of adult SC and other limbal vessels. (Left) DIC image of a whole mount prepared as in Figure S1. SC and limbal vessels are located in the limbus, just inside the dark pigmented band (see Figure S1). (Middle) Localization of the limbal vessels stained with endomucin in a Z-projection of confocal limbal stacks encompassing the entire whole mount. The whole-mount stacks were Z-depth color-coded (ICE LUT, see Figure S1). Color scale shows depth code colors of all structures stained with endomucin from inside (in) the eye to outside (out), with the SC being the most internal coded structure. (Right) Overlay of DIC and immunofluorescence image. (B) Higher magnification showing SC in relation to blood vessels of the LVP. BV, blood vessel; cc, collector channel. The blood vessels (magenta) are closer to the external ocular surface than SC (cyan). (C, D) Imaged whole mounts with the basement membrane marker collagen IV (COLIV) used to highlight SC in enface (XY) and conventional (XZ) orientations. (C) XY view; the icon in the lower left corner indicates the orientation of SC (also used in subsequent figures). The inner wall (IW) is closer to the reader, and the outer wall (OW) is away from the reader. (D) XZ view through the plane indicated by yellow lines in (C). In this XZ orientation, the lumen of SC (*) is evident between the COLIV-labeled inner and outer walls (compare to E). (E) Frozen section with COLIV labeling overlaid on its DIC image. The similarity between the XZ-represented whole mount and frozen section is clear. Note the characteristic bulbous undulations of the inner wall protruding into the lumen (arrowheads). Scale bar, (A) 500 µm, (B) 100 µm, and (C–E) 20 µm.

The ocular drainage structures are delicate and easily damaged. This has impeded studies of their development in unsupported tissue preparations. Surmounting these impediments, we developed a whole-mount procedure for the limbus and anterior portion of the eye. This procedure allows us to study the entire mouse limbus and SC in 3D (Figure 1 and Figure S1). This method permits high-resolution in situ analysis of not only the entire adult SC but also the developing SC at a cellular level, and in the context of all limbal vessels and tissue. It overcomes substantial shortcomings of analyzing conventional sections, including the study of limited tissue expanses and the difficulties of accurately interpreting data from thin sections. It allows vascular connections between vessels types to be directly visualized.

To clarify the orientation of the images presented throughout this article, we use a 3D-coordinate system to align the conventional view of the SC and limbus in sagittal section to whole-mount images (Figure 1 and Figure S1). Figure 1 and Figure S1 also introduce a depth coding procedure that is used in other figures, and relates tissue depths to the conventional view of SC. This procedure codes tissues in different colors based on their relative distance from the external ocular surface.

### Whole-Mounted SC Is Readily Visualized Using Endomucin and Collagen IV

Endomucin is an endothelial sialomucin. In humans, both BECs and LECs express it [21]. In contrast, in mice it is most highly expressed in BECs of veins and capillaries and expressed at low or undetectable levels in other mouse endothelial cell types including LECs [22],[23]. Immunostaining with an endomucin antibody shows that adult mouse SECs express this sialomucin at readily detectable levels, though often lower than that in BECs (Figure 1A and B). It is not detectable in mature mouse LECs (Figure S2). As far we are aware, this is the first report of endomucin expression in SECs.

Adult SC is evident as a flat vessel running along the length of the limbus. In cross-section, its width varies from 50–200 µm. The vascular, basement membrane marker Collagen IV is also robustly produced by SC, and it highlights the characteristic anatomy of its inner and outer walls (Figure 1C–E). When Z-depth is color-coded, SC codes as cyan to blue. A prominent vascular plexus runs parallel and superficial to SC (codes magenta to red, Figure 1A,B), and we call it the limbal vascular plexus (LVP). Branches of the LVP penetrate to the depth of SC with some connecting to SC as collector channels for AQH. Other vessels that exit the eye run at right angles to the LVP and connect with it. These vessels are often filled with red blood cells (RBCs) and are the episcleral vessels (not shown).

### SECs Express BEC Markers and Have Continuous Cell Junctions

**Figure 2:**
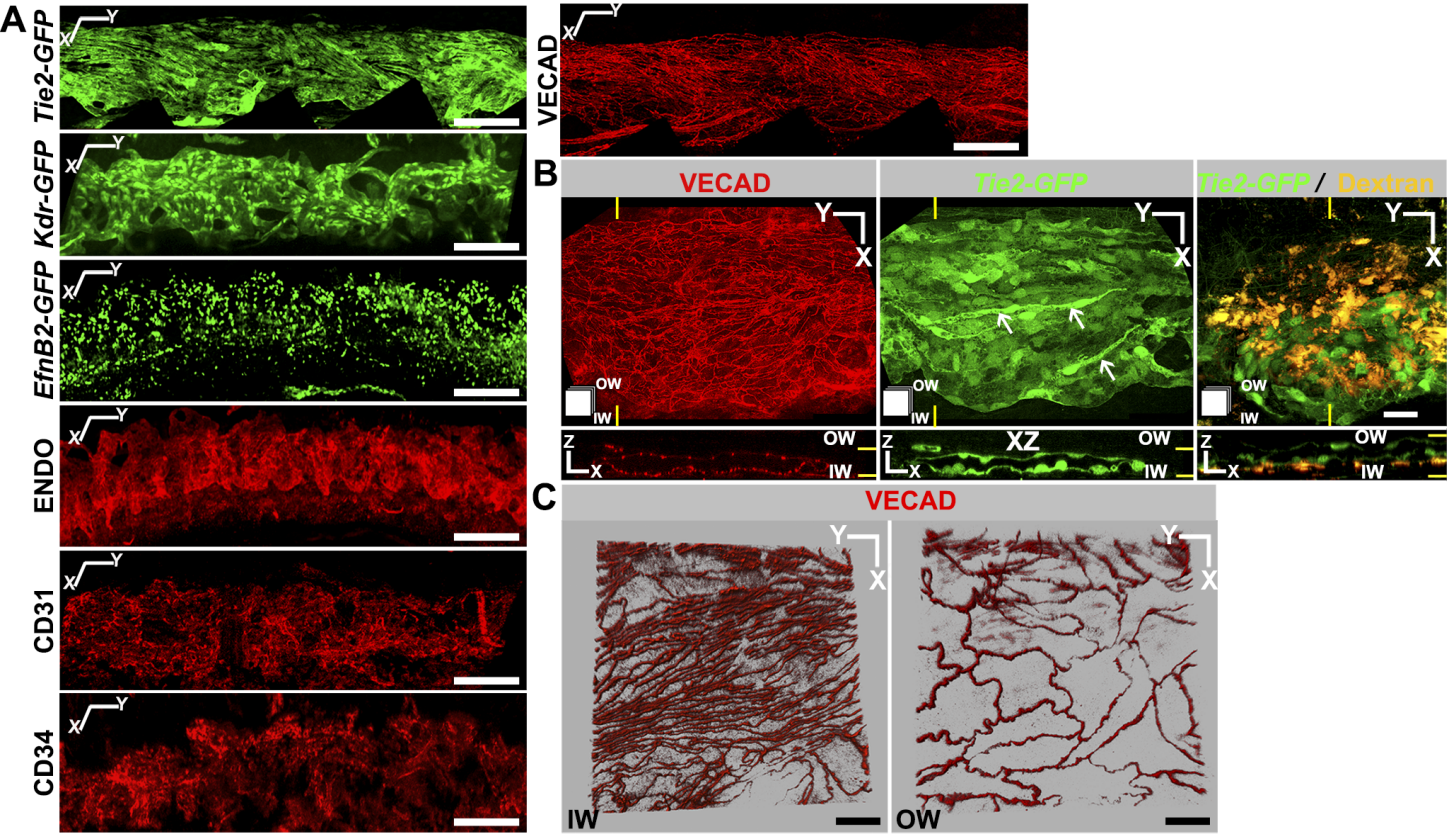
Adult SC expresses a panel of vascular markers. (A) Marker expression as determined by GFP expression (green, transgenic mice; see Methods) or immunostaining (red). Z-projections of limbal confocal planes encompassing SC. X and Y coordinate markers are slightly offset to indicate the curvature of SC. (B and C) Morphologically distinct cells comprise inner (IW) and outer walls (OW) of SC. (B) *Tie2-GFP^+^* SC immunostained with VECAD. Yellow lines indicate the locations of the XZ planes in bottom panels. Although *Tie2-GFP* continuously marks both walls, the cell junction marker VECAD is densely packed in IW but more widely spaced in the OW (bottom panels in B). This is consistent with IW cells being long (along Y axis) and thin (X axis) and OW cells being shorter and wider. The long thin nature of IW cells is clearly evident in the *Tie2-GFP* XY image (arrows). Confirming the identity of SC and its walls, fluorescent dextran that was perfused into the anterior chamber (yellow) accumulated at the IW (right panels). (C) Higher magnification images specifically showing IW and OW confirm this morphology. Z stacks encompassing either the IW SCE (Left) or OW SCE (Right) immunostained with VECAD were Z-projected in blend mode of Imaris to provide depth perception. Scale bar, (A) 100 µm and (B–C) 20 µm.

To facilitate studies of SC, and to further define the vascular markers expressed by it, we evaluated the expression of green fluorescent protein (GFP) transgenes controlled by vascular marker promoters and assessed other markers by immunolabeling. In addition to endomucin, SECs express a panel of vascular markers including *Tie2*, *Kdr* (*Vegfr2*), *EfnB2*, CD31 (PECAM1), CD34, and VE-cadherin (VECAD) (Figure 2A). Of these, CD31, CD34, and VECAD were previously identified in human SC [12],[24]. *Tie2* and CD34 are detected in BECs but not LECs under normal conditions [25],[26], whereas CD31 is expressed at much lower levels in LECs than BECs [25]. Resembling BECs, our data show robust expression of *Tie2*, CD31, and CD34 in SECs. KDR is an important vascular marker that is expressed in both BECs and LECs. VECAD is an adherence junction protein expressed in BECs and LECs and is critical in regulating junctional permeability [27]. VECAD localization between cells of blood vessels is continuous, contributing to an impermeable junction [28]. In contrast, VECAD has a discontinuous button-like localization in initial lymphatics, which are highly permeable [28]. In SC, VECAD localization resembles that of blood vessels and it serves as a useful marker for identifying SC with its distinct inner and outer wall morphology (Figure 2B and C). *Efnb2* is expressed in arterial endothelial cells and LECs but not in venous endothelial cells [29],[30]. Existing models suggest that SC is derived from veins and so expression of *Efnb2* may reflect differentiation towards a lymphatic phenotype. Our results thus show that the mouse SCE expresses common endothelial markers with important characteristics of BECs.

### SC Expresses Lymphatic Markers PROX1 and FLT4 But Not LYVE1

**Figure 3:**
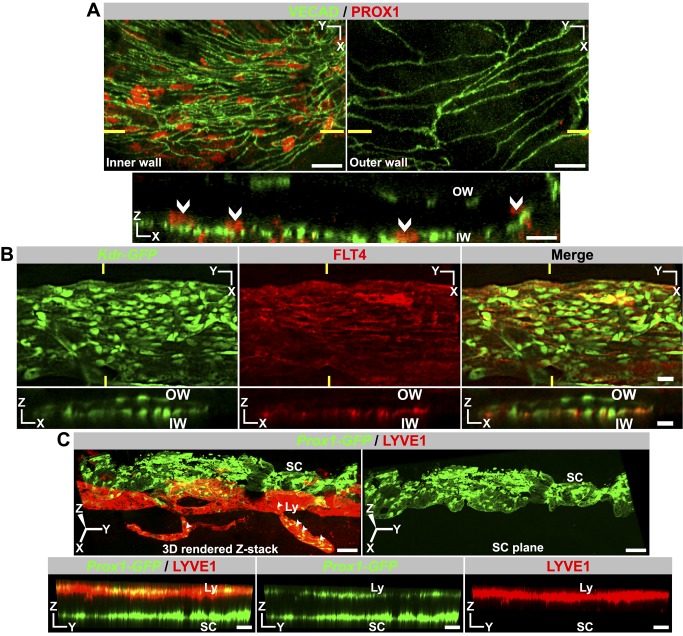
Adult SC produces the lymphatic markers PROX1 and FLT4 but not LYVE1. (A–B) Immunolabeling detects PROX1 protein and FLT4 in the inner wall (IW) SCE but not outer wall (OW) SCE. Nuclear localization of PROX1 is demonstrated in Figure S3. In both (A) and (B), high-magnification Z stacks that encompass either the IW or the OW were Z-projected to produce XY images (Top panel). XZ section (Bottom panel) through the plane indicated by yellow lines (Top panels) confirms expression of PROX1 is restricted to IW cell nuclei and FLT4 largely to the IW SCE. (C) LYVE1 protein is absent in SC. In the limbus, the lymphatics (Ly) run closer to the outer ocular surface than SC. In an enface 3D view from the outer surface perspective of a *Prox1*-*GFP* eye, the lymphatics run on top of the SC. (Left) 3D rendering has been rotated towards the viewer so that the lymphatics do not obscure the SC. The yellow represents the co-labeling of lymphatics with both LYVE1 and *Prox1-GFP* (arrowheads). (Right) The lymphatic in the left image has been removed, clearly showing that SC produces no detectable LYVE1. Bottom panels are the same YZ plane through the 3D rendering in the left top panel. Also see Figure S4. Scale bar, (A–C) 20 µm.

PROX1 and LYVE1 are commonly used as markers of a lymphatic lineage [31]. Given morphologic similarities between SC and lymphatics, we assessed expression of these molecules in SECs. Using immunostaining, we discovered that PROX1 expression is readily detectable in SECs of the inner wall but is not detectable in SECs of the outer wall (Figure 3A, Figure S3). Thus, PROX1, the master regulator of lymphatic phenotypes, may have a pivotal role in controlling the differentiation and maintenance of the functionally specialized inner wall SECs. To independently confirm *Prox1* expression and to provide a fluorescent tool for analyzing SC, we produced transgenic mice expressing GFP from the *Prox1* promoter (*Prox1-GFP*) [32]. Using these mice, we confirmed *Prox1* expression in inner wall SECs (Figure S4). Again, expression was predominantly in the inner wall, but low levels of expression were detectable in some outer wall cells (possibly due to less specificity of GFP expression from the transgene or greater sensitivity of this assay). These experiments indicate that the *Prox1* promoter is transcriptionally active in adult SECs with predominant expression in the inner wall cells. These results show for the first time that PROX1 is expressed in the SC, although at lower levels than in lymphatics. FLT4 (VEGFR3) is another key protein required for lymphatic development and also serves as a marker for lymphatics. FLT4 transcription is controlled by PROX1 [33]. Using immunostaining, we determined that FLT4 expression mirrors PROX1 expression, localizing predominantly in the inner wall of SC with low levels of expression in outer wall cells (Figure 3B). In contrast to PROX1 and FLT4, LYVE1 is not produced by SECs (Figure 3C). SC also does not express a commonly used lymphatic marker, podoplanin (Figure S5). Overall, the above data indicate that SC is a unique vessel with a blend of blood vessel and lymphatic phenotypes.

### SC Is Not Derived from Lymphatics

**Figure 4:**
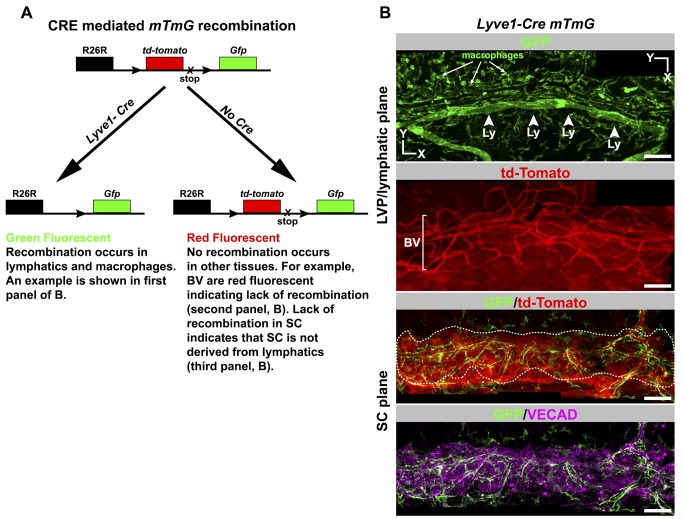
Lineage tracing using a *Lyve1-Cre* mouse shows SC does not originate from lymphatics. (A) Outcome of a genetic cross between a *Lyve1-Cre* mouse and the *Cre* reporter *ROSA26R-mtmG* (*mTmG*) mouse. *Cre* expressed from the *Lyve1* promoter is found early in developing lymphatic tissue and in macrophages. CRE-mediated recombination indelibly labels lymphatics and macrophages with GFP fluorescence in a backdrop of red fluorescent cells that are not of these lineages. Because this recombination is irreversible, tissue derived from *Lyve1*-expressing cells will always be green fluorescent. Cells that have never expressed *Lyve1-Cre* and whose ancestral cells never expressed *Lyve1-Cre* are red fluorescent. (B) SC does not originate from lymphatics. Corresponding confocal planes of adult SC at the levels of the labeled tissues are shown for a *Lyve1-Cre mTmG* mouse. The lymphatics (Ly, arrowheads) and macrophages (arrows) are green fluorescent, indicating *Lyve1-Cre*–mediated recombination occurred as expected. Blood vessels (BV) of the LVP and the corneoscleral tissue are red fluorescent, showing that *Lyve1-Cre* was never expressed in these tissues. SC cells are also red (GFP/tdTomato panel). SC is outlined by a dotted line to help distinguish it from the corneoscleral tissue that surrounds it. The form of SC is clearly demonstrated by VECAD immunolabeling (bottom). The green cells are macrophages associated with SC and not SC itself (see Figure S6). Scale bar, 100 µm.

Given limitations of previous studies and the earlier erroneous belief that the eye lacked lymphatic vessels, it remains possible that SC is derived from lymphatics. We used a *Lyve1-Cre* transgene [34] along with the *ROSA-mTmG* reporter gene [35] to indelibly label cells that are derived from LYVE1-expressing cells. In cells expressing both the *Lyve1-Cre* and *mTmG* transgenes, the CRE recombinase deletes a constitutively expressed *tdTomato* transgene flanked by *loxP* sites. This irreversibly activates expression of a *GFP* transgene, and this expression is passed on to descendent cells (Figure 4A). LYVE1 is first expressed in cells of the cardinal vein at embryonic day 9.5 and is the first indicator of these cells committing to a lymphatic fate [36]. Thus, the *Lyve1-Cre mTmG* mouse serves not only as an indicator of lymphatic expression but also of previous activation of a lymphatic developmental program. Our lineage tracing experiment clearly indicates that SECs and their progenitors do not express LYVE1 during development (Figure 4B and Figure S6). Thus, SC arises using a program different from embryonic lymphangiogenesis, and SECs are not derived from lymphatics.

### SC Is Not Derived from the Neural Crest

**Figure 5:**
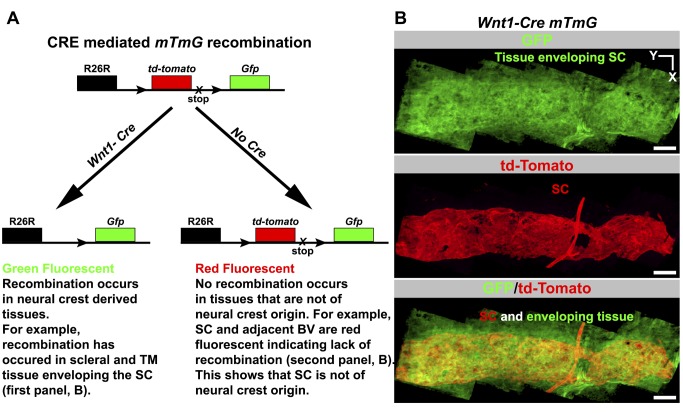
Lineage tracing using *Wnt1-Cre* shows SC does not originate from neural crest cells. (A) Outcome of a genetic cross between a *Wnt1-Cre* mouse and the *ROSA26R-mtmG* (*mTmG*) *Cre* reporter mouse. *Wnt1* is an early developmental marker for neural crest cells. CRE-mediated recombination indelibly labels neural crest-derived cells with GFP fluorescence in a backdrop of red fluorescent cells that are not of neural crest origin. TM, trabecular meshwork. (B) SC does not originate from neural crest cells. (Top) The tissues enveloping and adjacent to SC in *Wnt1-Cre mtmG* mouse. These tissues include corneoscleral tissue and TM. These tissue express GFP and are thus of neural crest origin. (Middle) Adult SC lacks GFP expression but expresses *tdTomato*, indicating that it is not derived from neural crest. VECAD labeling, shape, and location were used to identify SC (see Figure S7). A blood vessel connecting to the SC is also red fluorescent, showing BECs are not neural crest derived, as is true for all limbal vasculature (not shown). (Bottom) A merge of the top two panels. Scale bar, 100 µm.

For some time, it has been suggested that SC is derived from mesoderm but not neural crest cells [37]. More recently, this was supported in mouse eyes using *Cre*-based lineage tracing [38]. Because a lacZ reporter and conventional sections were used, a mixed contribution of progenitor cell types including some neural crest cells could have been missed. Thus, we have revisited the possibility of a partial neural crest contribution by analyzing the entire SC following lineage tracing using both *Wnt1-Cre* and *ROSA-mTmG* transgenes. Our results confirm previous findings and clearly demonstrate that neural crest cells do not contribute to SC development, even though the surrounding tissues have a strong neural crest contribution (Figure 5, Figure S7). Our data also confirm that neural crest cells do not contribute to the vasculature of the limbus (Figure 5), which is of mesodermal origin [38]. Together our results are consistent with a common mesodermal origin of both SC and the limbal vasculature, and they support a developmental model where SC arises from limbal blood vessels.

### SC Develops from Blood Vessels of the Limbus

**Figure 6:**
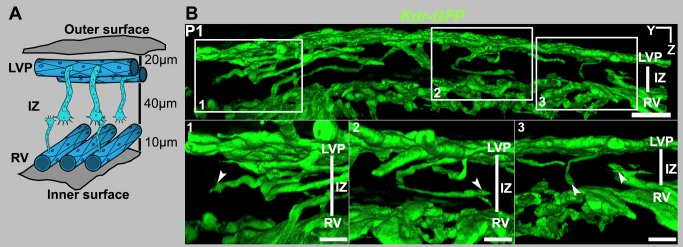
SC development initiates by sprouting from blood vessels in the limbus. (A) Cartoon representing an early stage in the development of SC. At P1 and P2, endothelial cells of both the LVP and the RVs form sprouts, which migrate into the zone where SC develops. Because this zone is between the LVP and RV, we have named it the “intermediate zone,” or IZ. The outer and inner surfaces of the wall of the eye are indicated. At this stage, the LVP circumscribes the eye superficially about 20 µm below the outer surface of the eye. The RV run radially around the eye approximately 60 µm below the outer surface of the eye and perpendicular to the LVP vessels. (B) Sprouts from the LVP and RV penetrating into the IZ. The top panel is a panoramic YZ view of a segment of the limbus from a *Kdr-GFP* eye at P1. GFP^+^ sprouts from both the LVP and RV are seen in the IZ. This panoramic view shows the entire tissue thickness. Boxed regions in the panoramic view are shown at a higher magnification in the three bottom panels. Note that the sprouts appear to have a leading tip cell and a trailing stalk-like structure (arrowheads). Scale bar, (top image) 40 µm and (bottom images) 20 µm.

Hypothesizing that SC develops from limbal blood vessels and revaluating the existing developmental models, we studied SC development in the context of all limbal vasculature. Because *Kdr* is expressed in both BECs and SECs, we used *Kdr-GFP* heterozygous mice [39] to visualize the limbal vessels and developing SC (*Kdr* heterozygous mice are established to have normal vascular development [40]). Moreover, we found that the SC of *Kdr-GFP* heterozygous mice develops normally when compared to C57BL/6J mice (e.g., Figure 2). We established that the LVP in a P1 eye is complex and runs completely around the eye just below the external ocular surface (Figure 6 and Figure S8). Deep within the limbus and close to the inner surface of the ocular wall there is another vascular bed. These vessels run radially in a direction perpendicular to the LVP. We named these vessels the “radial vessels” (RVs, Figure 6 and Figure S8). SC will form in the tissue between these vascular beds, a region that we name the “intermediate zone” (IZ). At this age, the IZ is ∼40 µm thick (Figure 6A and B). Signs of SC development are evident at P1, as endothelial sprouts penetrating into the IZ. Importantly, these sprouts emanate from both the LVP and RV (Figure 6B). Such sprouts are present all around the limbus and penetrate 10–20 µm into the IZ, the region where SC develops.

### Endothelial Sprouts Penetrate into the IZ Using Tip Cells

**Figure 7:**
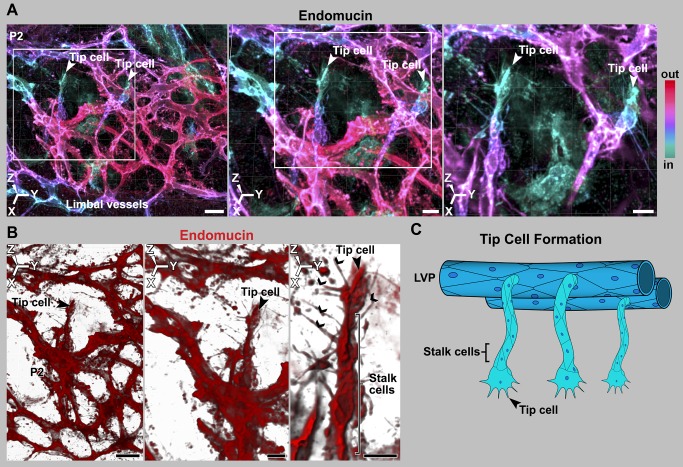
Sprouts have leading tip cells and following stalk cells, as is characteristic of angiogenesis. (A–B) Tip cells from the LVP penetrating into the deeper limbal tissue. (A) Depth coded maximum intensity 3D rendering of limbal region of a P2 eye stained with endomucin. The deepest, endomucin-positive tissue is colored cyan and the more superficial tissue is redder. (Left) Low-magnification image with tip cells emanating from the LVP. Their cyan color indicated that they are in the plane of the future SC. (Middle) The region bounded by the white box in the left panel at higher digital zoom. (Right) The region bounded by the white box in middle panel at higher digital zoom with the superficial layer electronically removed in Imaris to show the tip cells in greater detail. The color key indicates the depth code in relation to relative depth from the outside of the eye (In, closer to inside of the eye; out, closer to outside of the eye; see Figure S1). (B) (Left) Tip cell region from middle panel of (A) rendered using blend mode of Imaris. This provides density so that the characteristic tip cell filipodial structures are clearly evident. (Middle and Right) Progressively, the tip cell in greater detail. (C) Cartoon representing this stage of development but ignoring the RV and their sprouts for the sake of simplicity. This is in no way meant to suggest lesser importance of RV. Scale bar, (A and B, Left images), 20 µm and (A and B, middle and right images) 10 µm.

Tip cells are specialized endothelial cells that are critical in angiogenesis. They are present at the leading edge of vascular sprouts and lead the migration of endothelial cells from the parental vessel into the surrounding tissue. They are characterized by long filopodia (which integrate environmental cues) and a characteristic polar morphology. They are followed by other specialized endothelial cells know as stalk and phalanx cells that connect the sprout to the parent vessel [41]. Tip and stalk cells are not stable cell fates but shuffle and interchange status during the sprouting process [42]. We discovered that the leading cells of the sprouts that penetrate into the IZ have a characteristic tip cell morphology with long filopodia (Figures 6 and 7). For clarity, and because they give rise to SC, we call these cells “SC-tip cells.” It is worth noting that these sprouts form in the context of active angiogenic remodeling within the LVP (see Figure S11). Angiogenesis is initiated by interaction of VEGFA peptides with their receptor KDR. Here we show that KDR and its ligand VEGFA-164 localize to the developing SC at an age of active tip cell formation and interaction (Figure S9 shows VEGFA-164 localization).

### SC-Tip Cells Interact with Each Other, Forming Cell Clusters

**Figure 8:**
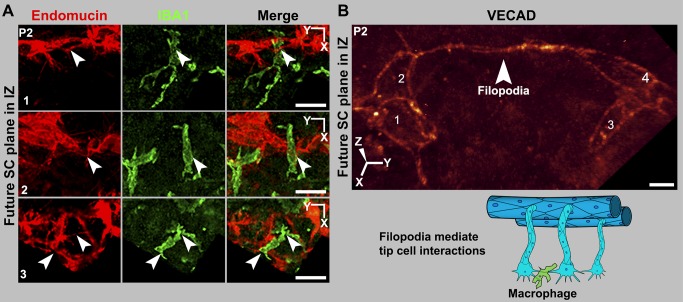
Macrophages are present at sites of tip cell interactions mediated by filopodia. (A) Tip cells in the IZ interact through their filopodia, and macrophages are present at sites of tip cell interaction. All images are Z-projections of the future SC plane of a P2 eye. The numbers correspond to their location in that eye in Figure S10. (Left) Endomucin staining of tip cells and their filopodia (arrowheads). (Middle) IBA1 staining marks macrophages. (Right) A merge of the endomucin and IBA1 staining. The interacting filopodia clearly have associated macrophages. (B) Interacting filopodia between two tip cells that are coated with VECAD. The filopodia are intertwined. The numbers label distinct cells. Image was captured using the highly sensitive photon-counting mode on the confocal microscope. The cartoon represents these tip cell–macrophage interactions. RV and their sprouts are not shown for the sake of simplicity and in no way meant to suggest lesser importance of RV. Scale bar, (A) 20 µm and (B) 5 µm.

**Figure 9:**
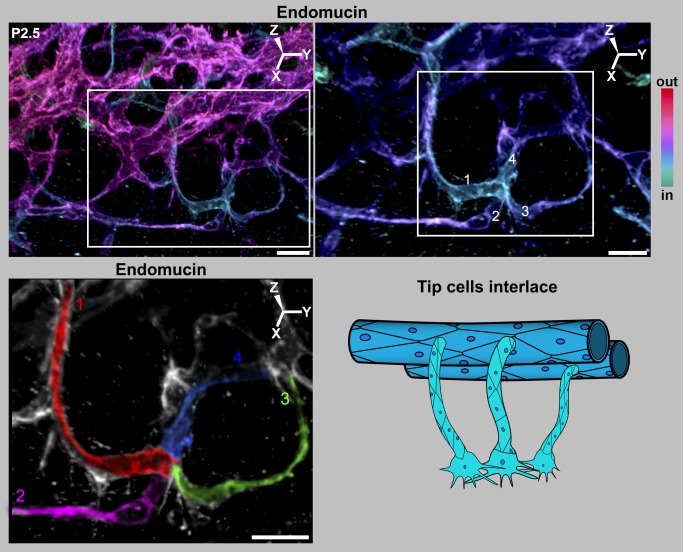
Filopodial interactions lead to interlacing of tip cells. (Left) Depth coded 3D rendering of limbal region of a P2.5 eye shows four tip cells interlacing with each other in the future SC plane. (Right) Magnification of the region within the white box in the left panel shows a cluster of interacting tip cells ranging in colors from cyan to violet. The complex LVP is in magenta. Superficial Z-slices were removed digitally in Imaris to reveal the interlaced tip cells with greater clarity. In this view, it is clear that each of the pseudocolored tip cells is connected to its own sprout and these sprouts connect to the LVP. Although the cell borders cannot be always unambiguously ascertained with endomucin staining, the fact that four individual sprouts are connected to this cluster clearly indicates that it originated from at least four tip cells. (Bottom) The sprouts and cells have been pseudocolored for clarity (numbered 1–4). The tissue that is not pseudocolored does not connect to this cluster. Cartoon represents the interlacing of tip cells. For simplicity, RV and their sprouts are not shown, and this in no way is meant to suggest lesser importance of RV. The 3D coordinates are shown. Scale bar, (Top Left) 20 µm and (Top Right and pseudocolored image) 10 µm.

**Figure 10:**
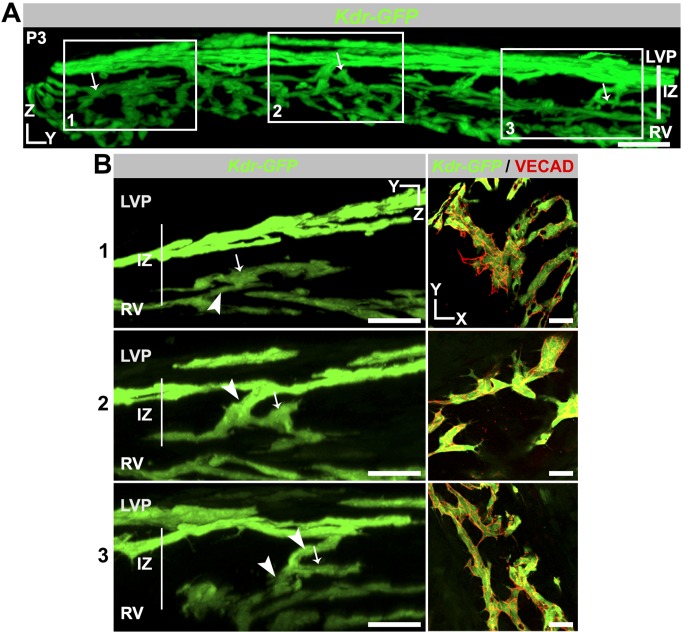
Cell clusters form from LVP and RV sprouts in the IZ. (A) Panoramic YZ view of a limbal segment of a *Kdr-GFP* eye at P3. GFP^+^ cell clusters that are still attached to LVP, RV, or both colonize the IZ. The clusters indicated by arrows occupy different tissue planes, and so the entire tissue thickness is shown. (B, Left) Three boxed regions from (A) at high magnification. For each boxed region and for clarity, tissue planes that obscured the view of the continuous connection between each cell cluster in the IZ and its originating vascular bed were digitally removed. Cell clusters connected to RV (1), LVP (2), and to both LVP and RV (3) are shown. Arrows are at identical positions in the panoramic and high-magnification views and indicate recognizable features of each cluster. Connections to parent vasculature are indicated by arrowheads. (Right) XY images of the same cell clusters show numerous GFP^+^ cells (green) attached to each other at cell junctions marked by VECAD. These cell clusters are the first multicellular sign of a nascent SC. Scale bar, (A) 50 µm and (B) 30 µm.

**Figure 11:**
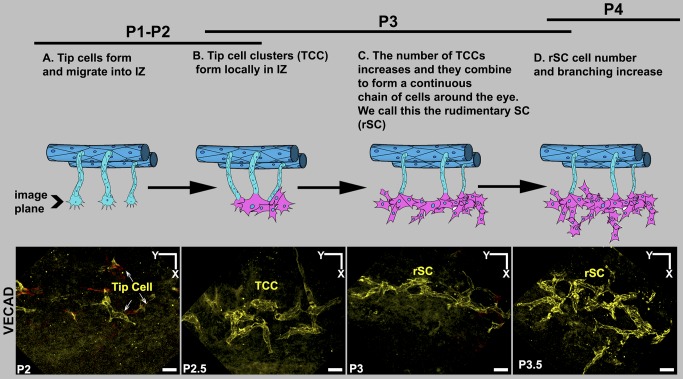
Summary of early SC development. The images show VECAD (yellow) immunolabeled cells in the IZ in the plane of the future SC at the developmental ages listed. The cartoons represent the corresponding stages. The development of SC starts by sprouting from the LVP and RV. RV and their sprouts have been left out of the cartoons for the sake of simplicity and in no way are meant to suggest lesser importance of RV. (A) As in angiogenesis, sprouts led by tip cells penetrate into the IZ all around the limbus. (B) However, in contrast to angiogenesis, multiple tip cells interact and form tip cell clusters (TCCs). (C) These local clusters of cells connect with each other to form a continuous structure encircling the entire limbus. We call this structure the rSC. (D) The number of cells in the rSC increases and the cellular chain branches. Scale bar, 20 µm.

In angiogenesis, pairs of tip cells interact with each other using their filopodia. Macrophages chaperone the filopodial interactions to accomplish anastomosis and the formation of a new vascular branch as the sprouts fuse [43]. Using endomucin and VECAD labeling of P2 eyes, we found that SC-tip cells interact with each other through their filopodia (Figure 8 and Figure S10). In most cases, the interacting filopodia have an attendant macrophage (arrowheads, Figure 8A). Thus, similar to angiogenesis (see Figure S11), macrophages appear to chaperone filopodial interactions of SC-tip cells. In angiogenesis, VECAD interactions between tip cells promote adhesion [44]. Thus, filopodial VECAD is expected to make adhesive contacts between SC-tip cells. In contrast to angiogenesis, where tip cells interact with each other and their attached sprouts anastomose, groups of SC-tip cells interact with each other without anastomosis of their sprouts and without new tube formation. Instead, the SC-tip cells adhere to each other and interlace in the IZ, while maintaining a flattened morphology and giving rise to tip cell clusters (Figure 9). By P3, the interacting SC-tip cells have formed tip cell clusters in the IZ throughout the limbus (Figure 10 and Figure S12). Many of these clusters are still attached to the LVP, RV, or both LVP and RV (Figure 10). However, not all of the cell clusters are connected to blood vessels. In these cases, it is not clear if connections to blood vessels were already pruned or if some of these clusters originated separately from other undefined progenitors. The number of tip cell clusters continues to increase. Between P3 and P4, the clusters combine with each other to form a continuous flattened chain of cells (Figure 11). Although this chain lacks any tubular morphology, we have named it the rudimentary SC (rSC) because it encircles the entire limbus. By P3.5 to P4, the number of cells in the rSC increases and it becomes more branched within its original tissue plane (Figure 11). This process resembles vasculogenesis in that cells divide and form branched chains prior to vessel formation. The rSC is still connected to the LVP and RV.

### rSC Regionally Expresses PROX1 and Forms Flattened Tubes

**Figure 12:**
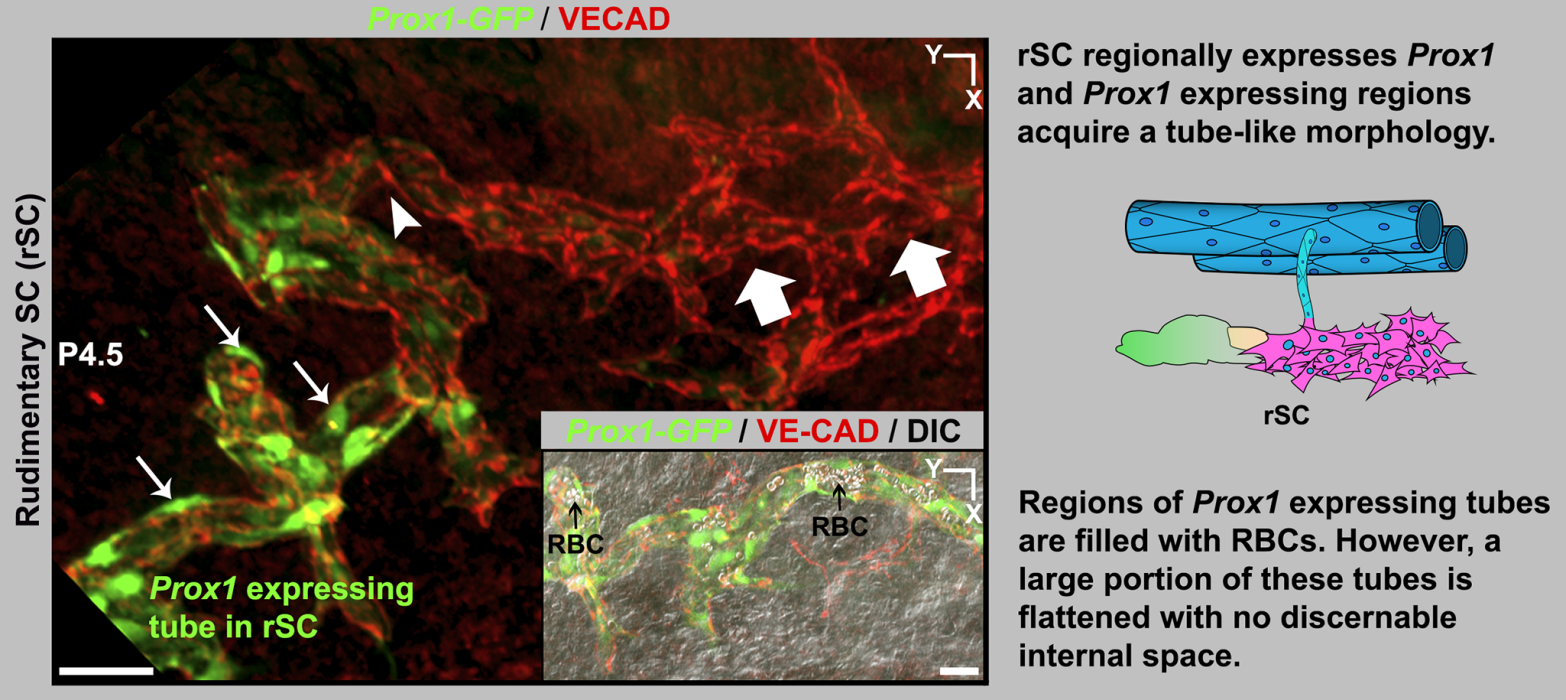
The rSC begins to express *Prox1* and to acquire a tube-like morphology. Z-projection of confocal stacks encompassing the rSC from a *Prox1-GFP* eye at P4.5. VECAD immunostaining shows a region with branching of the rudimentary vessel (block arrows, equivalent to stage D in Figure 11). *Prox1* is first expressed in the rSC at late P4. The branching rSC begins to acquire *Prox1* expression as indicated by GFP^+^ cells (arrowhead). Higher *Prox1* expression correlates with transition of rSC morphology from a sprouting chain of cells to a flattened tube (arrows). (Inset) DIC merged image showing that the tubular regions of the rSC have pockets of space filled with RBCs (black arrows). Scale bar, 50 µm.

As shown above, the adult SC expresses *Prox1*. Because *Prox1* is a potent developmental regulator, we have monitored its expression at all stages of SC development using the *Prox1-GFP* mice. During lymphatic development, one of the earliest detectable events is the activation of *Prox1* expression. Unlike lymphatic development, *Prox1* is not expressed at the earliest stages of SC development. The first detectable *Prox1* expression occurs regionally in the rSC at late P4 to early P5. *Prox1*-expressing regions are reorganized into flattened tube-shaped vessels (Figure 12). The level of *Prox1* expression increases with an increase in tubular morphology (compare region between arrowheads and arrows, Figure 12). At this stage, most of the tubes are flattened, but regionally they are filled with RBCs, reflecting continued connection to blood vessels (Figure 12). Thus, expression of *Prox1* correlates with the transition from a flattened chain of sprouting cells to a tube. Importantly, PROX1 expression remains lower than in lymphatics and is not easily detectable by immunostaining. As in the adult SC and discussed above, LYVE1 expression is absent in the developing SC.

### SC Increases in Size by Continued Sprouting and Cell Proliferation

**Figure 13:**
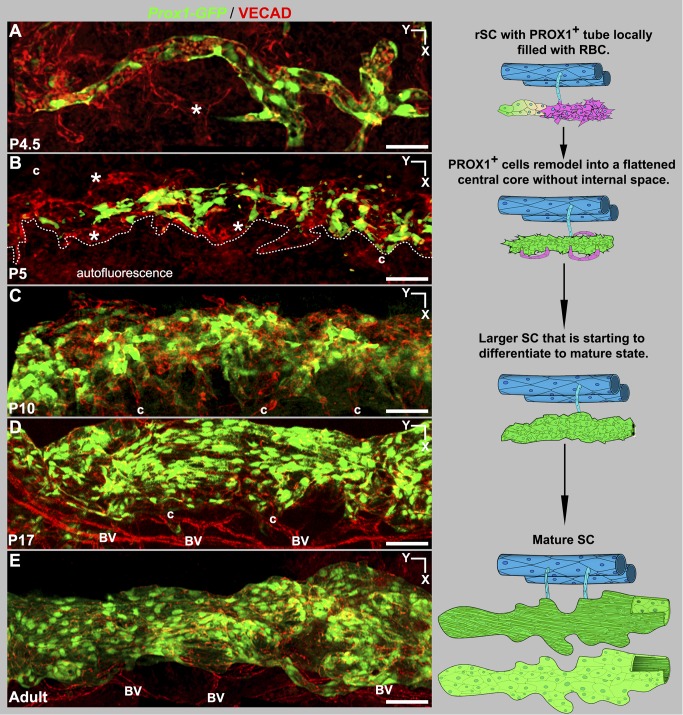
Development from rSC to mature morphology. (Left, A–E) Z-projections of confocal stacks encompassing the developing SC in *Prox1-GFP* eyes. (A) At P4.5 VECAD labeling shows multicellular sprouts (*) at the sides of the GFP + tubular rSC. These sprouts have no detectable *Prox1* expression. (B) By P5, remodeling has formed a central core of PROX1^+^ flattened cells without any discernible internal space or RBCs. Further development continues by sprouting (*, Figure S13) from the central core. Dotted line delineates developing SC from autofluorescence arising from nearby tissue. (C) At P10, developing SC has expanded considerably in size, and maturation with lumen formation and polarization of PROX1 expression to the inner wall have begun (see Figure 15). The developing SC remains connected to the LVP (c) but no connections to the RV are detected after P5. (D) SC looks mature at P17 but continues to grow. (E) Adult SC. (Right) The cartoons distill the essential points of each stage. At P5 PROX1^-^ sprouts are shown in magenta. At all developmental stages, the SC is connected to the LVP. The adult stage SC cartoon attached to the LVP is seen from the inner wall perspective, where the thin cells are a darker green to depict strong *Prox1* expression. The SC shown below is from the outer wall perspective, with the paler green large cells having weak or no detectable PROX1 expression. BV, blood vessels; c, collector channels. Scale bar, 50 µm.

**Figure 14:**
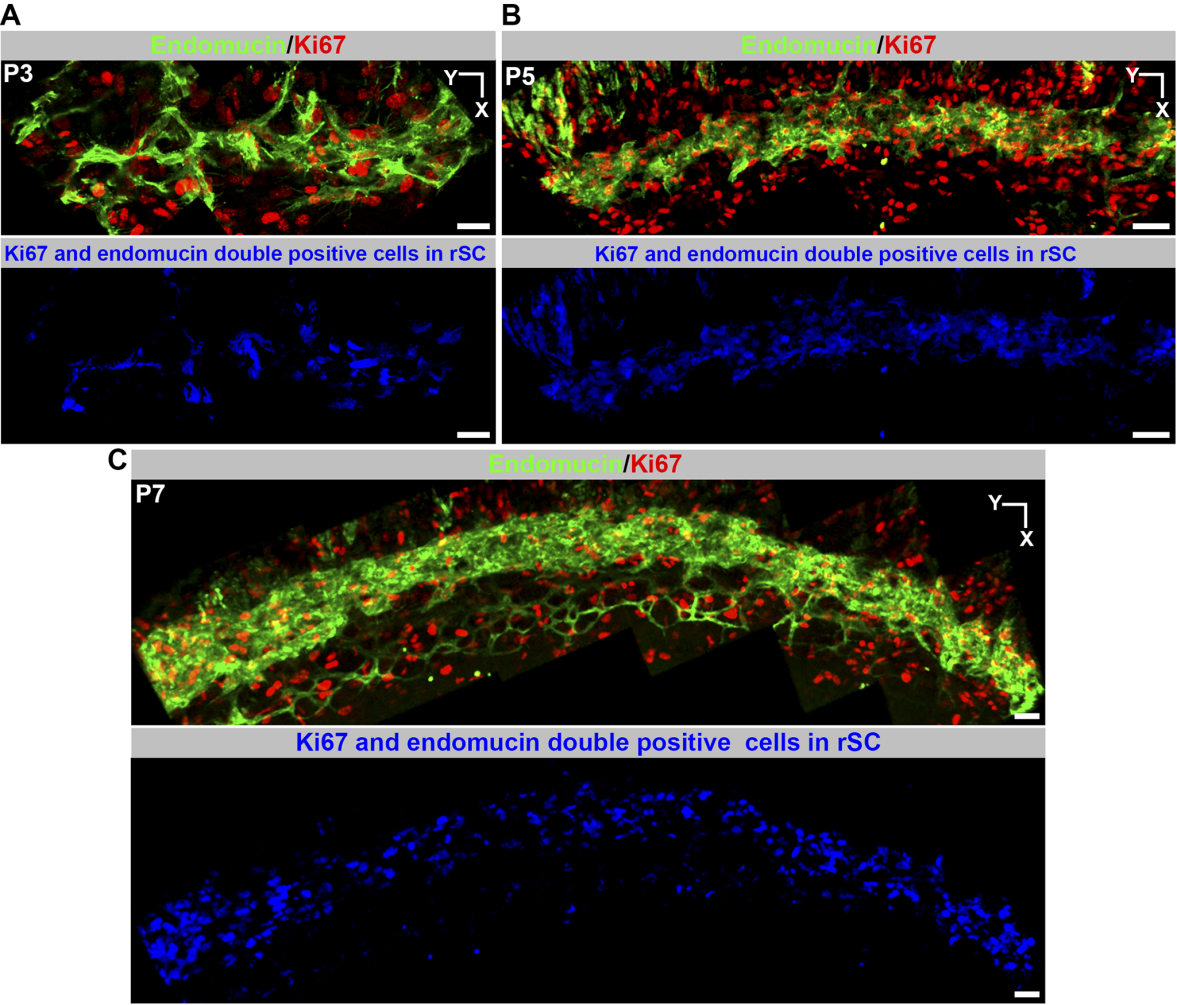
Cellular proliferation occurs during SC development. (A–C) Z-projections of the presumptive SC plane at the indicated ages. The top panels at each age show a merged image of endomucin and Ki67 staining. The endomucin staining delineates the developing SC. Ki67 labels dividing cells, labeling both the developing SC and other cells. Bottom panels show endomucin and Ki67 double-labeled cells in the developing SC in blue (voxel overlap of endomucin and Ki67 in a Z-projection). The double-positive proliferating cells closely track the developing SC. Thus, SC growth involves cell proliferation. Scale bar, 50 µm.

**Figure 15:**
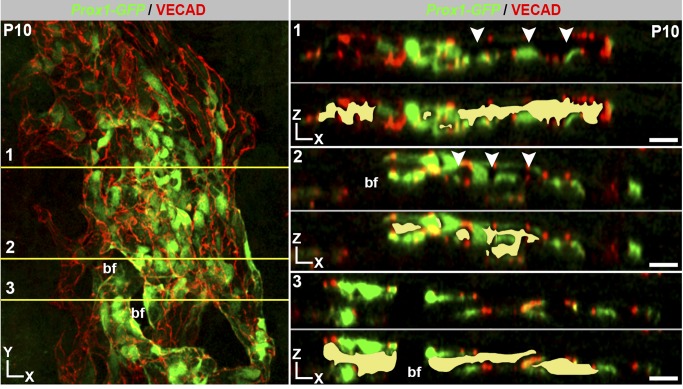
Polarized wall morphology and lumen formation are occurring at P10. (Left) Z-projection of high magnification confocal sections encompassing the developing SC in a *Prox1-GFP* mouse. (Right) XZ planes through the three regions numbered in the XY image. Each XZ plane is shown in its native state (above dotted line) and with a yellow fill marking regions with an obvious lumen (below dotted line). All sections are oriented with OW on top and IW on bottom. Varying degrees of lumen formation and maturation are evident. In 1, the lumen is almost complete and the IW and OW differentially express *Prox1*, with most of the OW no longer expressing detectable *Prox1* (arrowheads, compare to 2). In 2 and 3, the lumen is less continuous and differing portions of OW still robustly express *Prox1*. The complete breaks in labeling of IW and OW towards the left of 2 and 3 are due to a bifurcation (bf; compare to XY image). Scale bar, 30 µm.

The developing SC increases in girth with obvious growth until P17 and with subtler increases afterwards. The continued development and growth of SC involves sprouting from the rSC. Sprouts from rSC are first detected at P4.5. By P5, the PROX1-expressing (PROX1^+^) tubular regions have remodeled into a flattened core running centrally along the developing SC (Figure 13). By this stage, the flattened core has no detectable internal space and no longer contains RBCs. Sprouting continues and sprouts appear to loop back and connect to the main vessel or other sprouts. These sprouts do not end with an obvious tip cell but have long filopodia that are not restricted to their leading cell. Filopodia are also obviously evident on cells along the main body of the developing SC (Figure S13). This is different to both classic angiogenic sprouting and the initial sprouting into the IZ during SC development where filopodia are restricted to tip cells. The filopodia appear to be important for adhesion of sprouts to each other and to the main trunk of the developing SC. These sprouts lack detectable PROX1 expression. By P9 no sprouts are discernable. Cell proliferation, detected using the proliferation marker Ki67 [45], is also important to the growth of SC and is readily detected at assessed ages from P3 to P7 (Figure 14). Cell proliferation is not restricted to the sprouting regions. By P9 almost all cells express PROX1. At P10, lumen formation and differentiation to a mature cellular architecture is occurring at some locations, with loss of PROX1 expression beginning to be evident in outer wall cells (Figure 15). SC has a largely mature appearance by P17, but minor remodeling occurs later.

### SC Development Requires KDR Signaling

To begin to define the molecules necessary for SC development, we tested the functional importance of the tyrosine kinase receptor KDR (alias VEGFR2, a receptor for vascular endothelial growth factor, or VEGF). VEGF is an important signaling protein in both vasculogenesis and angiogenesis [46]. VEGF interacts with KDR on the surface of tip cells to elicit the angiogenic program [47]. To inhibit KDR function, we injected a specific rat-derived inhibitory antibody called DC101 that binds KDR and competitively inhibits VEGF signaling [48]. This antibody is a valuable inhibitory tool not only because of its proven specificity but also because homozygous genetic disruption of KDR results in embryonic lethality [40], whereas heterozygous disruption of KDR has no effect on SC development (see above and Figure 2). We injected DC101 and control antibodies into heterozygous *Kdr-GFP* mice. DC101 injection into these mice resulted in an almost complete absence of SC. However, this result is difficult to interpret because there was also serious disruption of the LVP and RV (not shown). Given this drastic effect on the LVP and RV, it may not be possible to use homozygous conditional gene knockouts of KDR without significant confounding effects on the vasculature from which SC arises.

**Figure 16:**
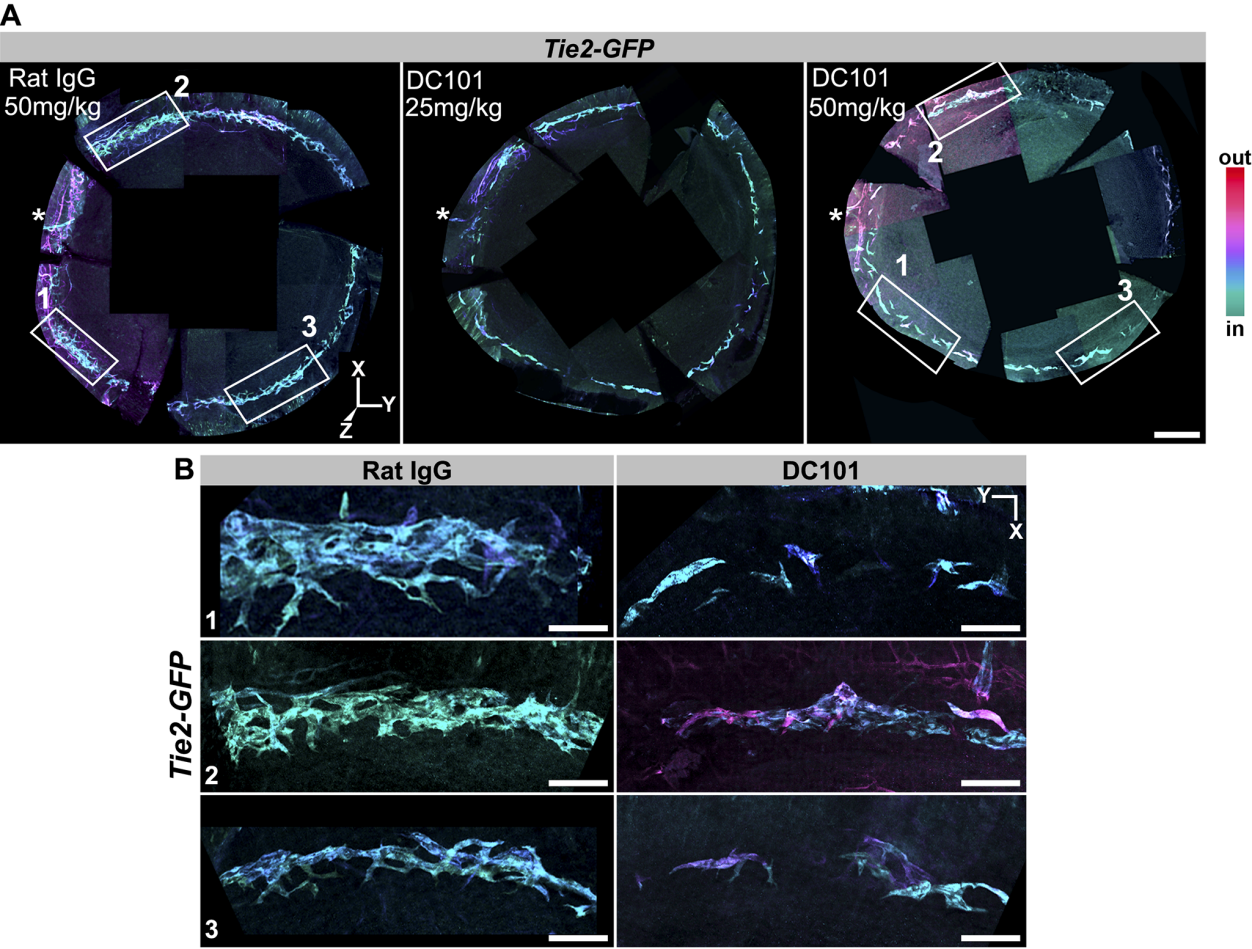
Blocking KDR function disrupts SC development at an early stage. (A) Whole mounts from *Tie2-GFP* mice injected with either a control (nonspecific IgG) or KDR (alias VEGFR2) inhibiting antibody (DC101). The antibodies were injected from P0 to P5 and the tissues studied at P6. Images are Z-projections of stitched confocal stacks encompassing the entire limbus. The images were oriented identically based on the location of a distinctive blood vessel that is always located close to the asterisk but outside of the images. GFP^+^ tissues in the stacks have been depth coded. SC is coded in shades of blue (cyan to violet). Elements of the LVP are hued magenta. (Left) Control. The control had no effect on SC development and is indistinguishable from untreated eyes. At this stage, SC has a braided appearance but variable robustness in different regions, as it has formed to different degrees around the eye. Although there is variability, it is typically robust in the boxed regions on the left in these images. It is often less distinct around the 2 to 4 o'clock position and in the region around 9 o'clock. (Middle) 25 mg/kg DC101. SC is generally less developed and appears more tube-like than braided, and it is more fragmented. (Right) 50 mg/kg DC101. SC development is profoundly affected. It is highly fragmented, appearing as low complexity cellular clusters. (B) Higher magnification of boxed regions in (A). Although the control has regional variation in width and robustness of braiding, the DC101 treatment results in substantial developmental retardation. Thus, KDR has an important role in early SC development. Scale bar, (A) 300 µm and (B) 40 µm.

**Figure 17:**
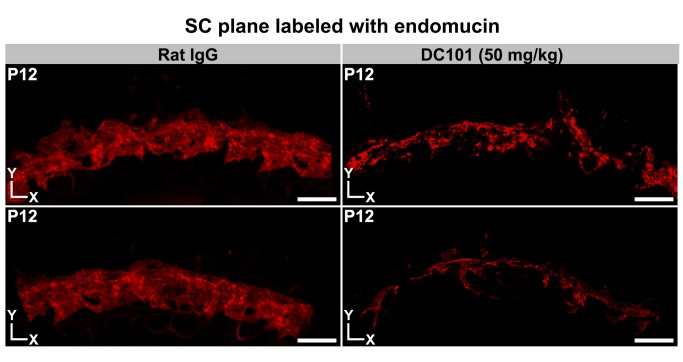
Blocking KDR function has a sustained disrupting effect on SC development. Whole mounts from P12 mice injected with the control (IgG) or inhibitory (DC101) antibodies. Images are Z-projections of confocal stacks encompassing the SC. Multiple Z stacks were first stitched together to obtain a panoramic view of a region of the whole mounts. (Left) Control. The control IgG-treated eyes were indistinguishable from untreated eyes, with a robust and complex morphology. (Right) DC101 treated. The KDR inhibiting antibody had a profound effect on SC development. Representative examples from different mice are shown. Scale bar, 100 µm.

To test the effects of KDR inhibition without these confounding complications, we tested two doses of DC101 in mice that have no mutations in KDR. DC101 was first injected into *Tie2*-*GFP* mice. KDR inhibition from P0 to P6 clearly disrupted SC development, with the higher of two doses of DC101 having the most profound effect (Figure 16). Injection of a nonspecific control antibody had no effect on SC. We next injected DC101 into C57BL/6J mice from P0 to P12. Again, KDR inhibition substantially impeded SC development, whereas the control antibody had no effect (Figure 17). Importantly these doses of DC101 had no discernible effect on the LVP (Figure S14) and RV (not shown). Injection of DC101 after P5 had no obvious effect on SC development, indicating that the critical window of KDR signaling is at earlier ages. These results clearly demonstrate that KDR signaling plays a critical role in the early development of SC.

## Discussion

**Figure 18:**
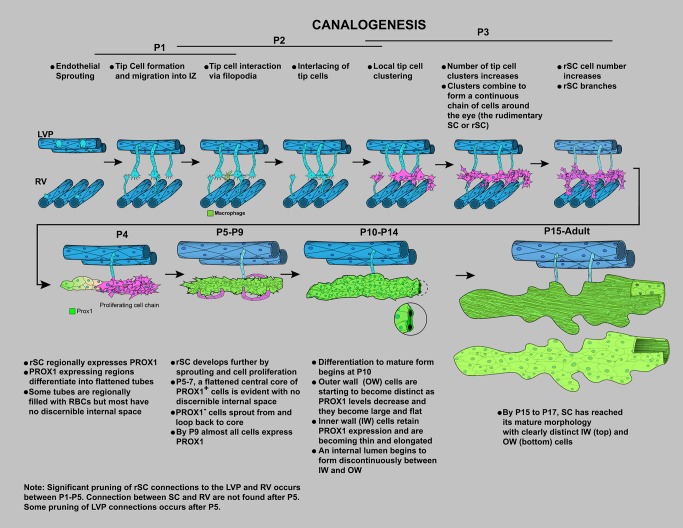
Schematic showing the stages of SC development by the novel process of canalogenesis. Cartoons have been drawn for clarity and are not intended to suggest that most early sprouts arise from the LVP.

SC is an important and highly specialized vessel but is poorly understood. Here we present new tools that will facilitate future studies of SC, and we present valuable information about its development and molecular phenotype. Importantly we discover that SC has a unique molecular phenotype and develops by a previously unknown sequence of vascular development that we name “canalogenesis” (Figure 18). SECs have features resembling both blood and lymphatic vasculature, and its endothelial cells have molecular similarities to both cell types. For the first time, we establish that both the developing and mature SC express the lymphatic master controller PROX1, which is likely of critical importance for inducing and maintaining key features of SC's functional specialization. We also demonstrate a critical requirement for KDR signaling early during canalogenesis.

Canalogenesis has similarities to three well-characterized developmental processes of vessel formation—namely, angiogenesis, vasculogenesis, and lymphangiogenesis. Although early steps of canalogenesis have key similarities to angiogenesis, including endothelial sprouting and tip cells, later steps of canalogenesis are distinct. In angiogenesis, tip cells, at the end of endothelial sprouts, interact together resulting in anastomosis of sprouts producing patent, vascular branches. During canalogenesis, however, several tip cells interact and adhere together to form clusters of tip cells without either anastomosis or formation of a new tube. Cells in these clusters divide, producing a chain of cells prior to lumen formation. This process resembles stages of vasculogenesis, where endothelial cell clusters derived from angioblasts form a chain of cells [49]. In both canalogenesis and vasculogenesis, the chain of cells forms a tube (a new capillary in the case of vasculogenesis). However, unlike vasculogenesis, PROX1 expression is activated in the endothelial cell chains during canalogenesis, and the PROX1^+^ cells remodel to form a tube. The branching of cellular chains that acquire a tube-like morphology also has similarities to the formation of the lymph sac during lymphangiogenesis [50]. Although the branching cells are PROX1^−^ in canalogenesis, they are PROX1^+^ in lymphangiogenesis. Thus, the timing of PROX1 expression differs between these processes. Canalogenesis also differs from lymphangiogenesis in that LYVE1 is not expressed in the developing SC. Thus, canalogenesis combines elements of angiogenesis, vasculogenesis, and lymphangiogenesis but is significantly different from any one of them.

At a molecular level, we have identified an early and critical requirement for KDR functions during SC development. Localization of VEGFA-164 to the developing SC indicates that this molecule is a ligand for KDR (as it is in angiogenesis). Because KDR is expressed in the adult SC, VEGFA may regulate SC permeability or mediate other functional roles. This is consistent with a recent report that mice heterozygous for mutations in both *Kdr* and *Flt1* (*Vegfr1*) have elevated IOP [51]. Together with our study, this suggests that mutations in genes coding for proteins critical for SC development will cause high IOP and increase the risk of glaucoma. At this point, it is unclear if other VEGF receptors such as FLT1 (VEGFR1) and FLT4 (VEGFR3) play a role in development of the SC. Of these, FLT4 is important for lymphatic development and is a key marker of mature lymphatics. FLT4 expression is activated by PROX1. Therefore, FLT4 may play a functional role in SC development. Importantly and supporting this, we demonstrate that FLT4 is expressed in SC with its expression mirroring the polar distribution of PROX1 (greater in the inner wall compared to the outer wall of SC). Thus, the FLT4 ligands VEGFC and VEGFD may play key roles in SC development and maintenance/adult function. SC also expresses the angiopoietin receptor TIE2. The TIE2/angiopoietin pathway is crucial in angiogenesis, lymphangiogenesis, blood vessel maturation, endothelial health, and regulating vascular homeostasis [52]. Thus, TIE2 and angiopoietins could play a role in both SC formation and regulation of SC permeability.

Recently, the *Caenorhebditis elegans* homologue of PROX1 (PROS-1) was shown to play a role in formation of the tubular excretory canal [53]. Given the concurrence of PROX1 expression with tube formation in the developing SC, it seems possible that PROX1 plays an important role in tubulogenesis in the rSC. The triggers for *Prox1* expression remain unclear. *Prox1* expression could be triggered by mechanical stimuli, as yet unidentified hemodynamic changes, cell morphologic changes, or cell–cell interactions in the branching rSC. Recently, both cell–cell interactions and mechanical stimulation by fluid flow have been shown to regulate the expression of *Prox1* [54]–[56]. Thus, AQH outflow may be a factor maintaining PROX1 expression in the adult SC and at developmental stages when it functionally drains AQH. It does not seem possible that AQH flow initially induces PROX1 expression during P4, however, as the trabecular anlage remains a compressed mass of cells [57]. Even if the still developing ciliary body [57]–[60] produces AQH at this stage, this cellular mass would not allow flow of this fluid to the developing SC.

Although there are additional components, our findings are in line with earlier models from human and mouse tissues, where SC develops from superficial blood vessels that penetrate deep into the sclera [18],[19]. A significant addition is that we establish a significant contribution to SC from two vascular beds, the superficial LVP and the deep RVs. In agreement with our previous study [19], we show that lumen formation in the developing SC occurs discontinuously and initiates at P10. Unique features of SC development are the polarization of *Prox1* expression to the inner wall compared to the outer wall and the accompanying differentiation of the unique cell types in each wall. We are unaware of any other vascular-derived tubular structure developing this way.

In adult SC, PROX1 is enriched in the inner wall cells and is likely important for its high degree of functional specialization. Inner wall cells are long and thin and readily deformed by pressure changes in the eye, likely reflecting specialization for mechanical sensing. The flow of AQH subjects the SECs to shear stress, especially near collector channels, which connect SC to the venous system. PROX1 is thus ideally positioned in inner wall cells to act as a regulator of AQH flow. PROX1 may control junctional permeability of the inner wall by controlling expression of VECAD as it does in lymphatic vessels [61]. PROX1 also regulates responses to fluid shear stress in LECs and is linked to mechanosensory signaling [54]. Modulation of PROX1 by pressure-dependent shear forces/strain could modulate the expression levels of downstream genes such as VECAD or alpha 9-integrin, which are produced by the inner wall cells of SC (unpublished results). This mechanism would allow SC permeability or cell adhesion to be responsive to pressure changes in the eye.

We also demonstrate that endomucin, a protein that plays a role in leukocyte rolling and is an early marker of vascular endothelial cells, is expressed in SC cells [62],[63]. Other vascular cell surface molecules such as CD34, CD31 are also expressed in SECs, and at levels similar to those in BECs. Along with other cell surface proteins, these molecules may modulate the behavior of immune cells that interact with SECs as they exit the eye. Thus, these molecules may modulate immune tolerance associated with the eye. Immune cells have been implicated in glaucoma and may mediate early neural damage [64]–[66]. It is possible that in addition to its influence on IOP, SC may influence glaucoma by modulating immunity.

In summary, this study provides novel insights into SC, clearly demonstrating that it is a unique vessel with a combination of blood vascular and lymphatic phenotypes, which develops by a previously unknown sequence of vascular developmental events. Overall, it provides new molecular insights and new tools that will greatly facilitate our understanding of the complex functions of this important canal.

## Materials and Methods

### Mouse Strain, Breeding, and Husbandry

This study was performed in strict accordance with the recommendations in the Guide for the Care and Use of Laboratory Animals of the National Institutes of Health. All of the animals were handled according to approved institutional animal care and use committee (IACUC) protocols (#99108) of The Jackson Laboratory. In addition, all experiments were conducted in accordance with the Association for Research in Vision and Ophthalmology's statement on the use of animals in ophthalmic research. All mice were housed in a 14-h light to 10-h dark cycle under previously described conditions [67]. The Jackson Laboratory's Institutional Animal Care and Use Committee approved all procedures described here. *Prox1-GFP* BAC transgenic mouse sperm (Tg(Prox1-EGFP)KY221Gsat/Mmcd, cryo-archived) [32],[68] was purchased from the Mutant Mouse Regional Resource Centers (MMRRC, UC Davis). A colony of these mice was established in our laboratory following rederivation by in vitro fertilization of C57BL/6J oocytes. The following mice were obtained from the Jackson Laboratory repository: Stock TgN(Tie2GFP)287Sato/J (JAX stock no. 003858) [69], Stock KDR ^tm2.1Jrt^/J (JAX stock no. 017006) [39], B6;129P2-Lyve1tm1.1(EGFP/cre)Cys/J (JAX stock no. 012601) [34], B6;129P2 (Cg)- Gt(ROSA)26Sor^tm4(ACTB-tdTomato,-EGFP)Luo^/J (ROSA mtmG, JAX stock no. 07676) [35], and STOCK Tg (Wnt1-cre)11Rth ZTg(Wnt1-GAL4)11Rth/J [70] (JAX stock no. 003829). The eyes from B6;129S4-EfnB2^tm2Sor^/J mice [71] were obtained from Dr. Taija Makinen, Upssala University. Pups were dated based on visualization of vaginal plugs in females in timed pregnancy crosses and also by closely monitoring the cages thereafter for newly born pups. In all, at least 20 eyes were analyzed for each development stage. As image collection is very time-intensive, we did not collect images from all eyes. Images of either the entire limbus or partial limbal regions were collected for at least 8–10 eyes for each developmental stage. Although the mice were of different strain backgrounds, the details and developmental timing of SC formation were not different between backgrounds. The strain backgrounds were: C57BL/6J (B6), ICR/HaJ, FVB/N, and FVB/N X B6. Adult SC was analyzed in >20 wild-type eyes and approximately 10 eyes with each *GFP* reporter gene genotype. For lineage tracing, six eyes were analyzed for both the *Lyve1-Cre* and *Wnt1-Cre* experiments, respectively.

### Anterior Eye Cup Whole Mounting

Enucleated adult eyes or heads of postnatal mice were fixed overnight at 4°C in 4% paraformaldehyde (PFA, Electron Microscopy Science, Hatfield, PA) prepared in phosphate buffered saline (1× PBS, 137 mM NaCl, 10 mM phosphate, 2.7 mM KCl, pH 7.4). The postnatal eyes were dissected out following fixation. The anterior part of the eye was cut just posterior to the limbus (see Figure S1), and the iris, lens, ciliary body, and thin strip of retina were carefully removed to obtain the anterior eye-cup. The anterior cup includes the cornea, limbus, and small potion of retinal pigmented epithelium. Four centripetal cuts were made to relax the eye-cup and facilitate eventual mounting onto a slide after immunofluorescence.

### Immunofluorescence and Confocal Microscopy on Anterior Eye-Cups

#### Immunofluorescence

Anterior eye-cups were washed with 150 mM glycine-0.1% Tween 20 in PBS for 5 min to quench residual PFA and then rinsed multiple times with 1× PBS. The eye-cups were incubated with 3% bovine serum albumin and 1% Triton X-100 in 1× PBS (blocking buffer) at room temperature for 1 h in 2 ml glass vials to block nonspecific binding of antibody and to permeabilize the tissue. The anterior cups were then incubated with primary antibodies of choice in 200 µl blocking buffer for 2 d, with rocking, at 4°C. The anterior cups were then washed three times over a 3-h period with 1× PBS containing 0.1% Tween-20. The primary antibodies were detected with the appropriate species-specific secondary antibody (all Alexa 488, 594, or 647 at 1∶1,000 dilution, Life Technologies, Grand Island, NY) diluted in blocking buffer, which also had DAPI to label nuclei. The immunostained eye-cups were washed four times over a 3-h period in 1× PBS. Eye-cups were then whole-mounted on slides in Fluoromount (Sigma, St. Louis, MO). The following primary antibodies were used in this study: Rat IgG against VE-cadherin (eBioBV13), endomucin (eBioV. 7C7), Ki67 (SolA15), podoplanin (eBio 8.1.1), and LYVE1 (ALY7) were obtained from eBioscience (San Diego, CA). All these antibodies were used at a 1∶100 dilution of the stock. Goat anti-LYVE1 antibody and goat anti-VEGFR3/FLT4 were obtained from R&D Systems (Minneapolis, MN) and used after 1∶100 dilution of the stock. The Collagen IV (134-01) goat antibody was from Southern Biotech (Birmingham, AL) and was used after diluting the stock 500-fold. The PROX1 antibody obtained from AngioBio (Del Mar, CA) was used at a 1∶200 dilution of the stock. Frozen sectioning, paraffin sectioning, and hematoxylin eosin staining were done as described in earlier studies [72].

#### GFP imaging

While imaging the *Prox1-GFP* SC, we noted that at all stages of development, the distribution and amount of the GFP marker varied within SECs. Sometimes it marks the entire cell, but other times it is more restricted to the nucleus. Cell expressing any trace of GFP were considered to be PROX1^+^. In addition, the cells expressing *EfnB2* were identified by nuclear fluorescence in the *EfnB2-GFP* transgenic, where the GFP is fused to histone H2B [71]. To ascertain there were no changes in *Kdr-GFP* mice, SC was stained with endomucin and compared to B6 SC. At least six eyes were analyzed and the limbus partially imaged.

### Confocal Microscopy

Microscopy was performed using an LSM SP5 or SP8 confocal microscope (Leica) using either a 20×0.7 NA multi-immersion objective or 63×1.4 NA glycerol immersion objective. The Mark and Find mode was used to automate collection of images encompassing the entire limbus and generated a folder full of Z stacks at various individual overlapping positions along the limbus. The SP8 confocal was also used when specialized settings such as the highly sensitive photon counting mode was required.

### Postprocessing of Images

#### 3D rendering

Individual confocal Z stacks (.lsm files) were processed directly using Imaris 7.6 (BitPlane AG, Zurich, Switzerland). Either the maximum intensity projection setting or the blend setting in Surpass mode of Imaris was used for 3D rendering. Controls and experimental image sets were treated identically. Images were oriented so that structures of interest were visible. Multiposition Z stacks generated using the Mark and Find setting on the confocal microscope were first imported into Imaris and converted into Imaris files (.ims files). The resulting Imaris files were then stitched to generate a comprehensive Z stack encompassing the limbus using XuvTools [73]. The stitch was exported as an Imaris file and processed appropriately. When making figures, the snapshot feature of Imaris was used to collect images at high resolution (1024×1024 pixels, 300 dpi). When appropriate, planes encompassing specific regions such as the SC or LVP were projected after eliminating extraneous planes by using either the Crop 3D or clipping plane functions of Imaris.

#### Orthogonal images

The Section view of Imaris allows one to visualize a Z stack from the XY, XZ, and YZ perspective all at once. To obtain specific orthogonal regions (XY, XZ, or YZ), a cross-hair can be placed at a point of interest in any of the three perspectives to obtain a plane along the cross-hair lines. In Expand mode, the cross-hairs can be expanded to provide a thick slice rather than a single plane. Expand mode can also be used to select subregions in a stack such as the SC plane.

#### Z-depth coding

Depth coding Z stacks was performed using the Z-code stack plugin for FIJI [74]. Multichannel confocal stacks were imported into Imaris. The appropriate channel displaying the marker of interest was extracted by deleting other channels. Z-depth coding was initiated by using FIJI functionality within Imaris. Z-depth coding assigns different colors to the individual planes of a Z stack. We have used the ICE lookup table (LUT), where colors range from cyan to red. In this coding scheme, the first plane of a stack is colored cyan and the last plane red.

### Functional Studies Using Antibody Inhibition

For inhibition of KDR signaling in vivo, the anti-KDR function-blocking rat monoclonal antibody DC101 (6.33 mg/ml, BioXCell, West Lebannon, NH) [48] or control rat IgG antibodies (Jackson ImmunoResearch) was injected subcutaneously as previously described [75]. The antibody was injected for a dose of either 25 or 50 mg/kg using a 33-gauge needle and Hamilton syringe. For most experiments, the antibody was injected daily at approximately 9 AM during the following periods: P0–P5, P0–P11, or P6–P11. For P0–P11 injections, 20 wild-type eyes were analyzed and complete or partial image sets of the limbus were collected for 6–8 eyes for each treatment and time point. Six wild-type eyes were analyzed for each of the P0–P5 and P6–P11 injections. For the *Kdr-GFP* heterozygotes, 12 eyes were analyzed up to P12.

## Supporting Information

Figure S1 Orientation, depth coding, and whole mounting. (A) Sagittal section of the adult mouse eye stained with hematoxylin and eosin. The dotted line indicates the plane through which the front of the eye is separated from the posterior segment during dissection. XYZ coordinates are shown to provide orientation for all other figures throughout the article. (B) Higher magnification of the limbal region (red box in A), demonstrating the relative locations of trabecular meshwork (TM) and SC drainage structures in the iridocorneal angle. C, cornea; RPE, retinal pigmented epithelium; Ch, Choroid. The color scale indicates the relative color of depth-coded tissues in relation to the external surface of the eye in Figure 1 and all other figures where depth coding is used. Superimposing this scale on this conventional sectional view provides orientation for the location of SC and other structures in the 3D enface views elsewhere in the article. Note that redder colors represent tissues closer to the surface of the eye, whereas the cyan coded tissues are closer to the TM. SC is adjacent to the TM and due to mild local variation in its tissue depth color codes in blue to cyan. The blood vessels (BVs) that comprise the LVP are closer to the ocular surface than SC and depth code as magenta (see Figure 1B). (C) Whole mounting procedure. The eye is enucleated and the anterior eye cup dissected away along the cutting plane. Next, the lens, iris, and ciliary body are removed from the anterior eye cup. Centripetal cuts are made to relax the cup so that it lies flat. Red brackets indicate the location of the limbus and provide further orientation in relation to (B). The dark band around the periphery of the whole mount represents the pigment of the RPE, which remains due to the plane of separation of the cup from the rest of the eye. For all XYZ coordinates, the wedge indicates the dimension into and out of the plane of the paper, while the other coordinates are in the plane off the paper. Scale bar, (A) 1 mm and (B) 100 µm.
(TIF)

Figure S2 Endomucin is not expressed in lymphatic vessels. Z-projection of confocal planes encompassing the LVP and lymphatics show that endomucin (Middle) is robustly expressed in blood vessels but not lymphatics labeled with LYVE1 (Left). Position of the lymphatics is outlined in the middle image. BV, blood vessels; Ly, lymphatics. Scale bar, 100 µm.
(TIF)

Figure S3 PROX1 expression in inner wall SCE. High-magnification confocal Z stacks of inner wall SCE rendered in 3D. Blend mode was used to give depth perception. The nuclei of VECAD-positive IW cells co-label for PROX1 (immunolabeling) and DAPI. As is well established, the DAPI-labeled nuclei bulge into the lumen of SC (towards the reader). Scale bars, 15 µm.
(TIF)

Figure S4 Primary localization of PROX1 in the inner wall of SC is confirmed using the *Prox1-GFP* transgenic mouse. (A and B) *Prox1* expression is enriched in inner wall SCE. (A) High-magnification Z-projection of confocal planes encompassing SC in a *Prox1-GFP* mouse eye immunostained with VECAD. The projections show that the characteristically long and thin IW cells robustly express *Prox1-GFP*. XZ sections show enrichment of *Prox1*-GFP expression in the IW compared to OW (bottom images). The IW is easily distinguished by the close-packed VECAD puncta at cell junctions. In comparison, GFP fluorescence in the outer wall (well-spaced VECAD puncta) is weak. XZ sections are through the plane indicated by yellow lines in XY panels. (B) Z-projection of confocal planes encompassing either the IW (Left) or OW (Right) labeled with VECAD and DAPI confirm enrichment of *Prox1*-GFP expression in the thin IW cells versus wide OW cells. Note that the GFP label varies in its intracellular localization, sometimes being primarily in the nucleus and other times throughout the cell. Scale bar, (A–B) 20 µm.
(TIF)

Figure S5 Podoplanin is not expressed in the SC. In an enface 3D view from the outer surface perspective of a *Prox1*-*GFP* eye, the lymphatics run on top of SC. In the top and middle images, co-labeling of podoplanin (PDPN) and *Prox1-GFP* renders the lymphatic vessel (Ly) a yellow color. *, lymphatic cells that have sheared off from the main vessel. SC is green as it expresses only *Prox1-GFP* but no podoplanin. Top image, 3D rendering showing the entire thickness of the limbal tissue has been rotated towards the viewer so that the lymphatics do not obscure SC. Middle image, Z-planes encompassing the lymphatic vessel. Bottom image, Z-planes encompassing the SC, Bottom image, podoplanin is not expressed in SC cells. Scale bar, 30 µm.
(TIF)

Figure S6 LYVE1 and IBA1 immunostaining confirm that the GFP^+^ cells associated with SC are macrophages. (A–B) Corresponding confocal planes at the levels of the indicated tissues from *Lyve1-Cre mTmG* mice. (A) Green fluorescent lymphatics (arrowheads, top image) and macrophages around them (arrows, top image) also immunostain with a LYVE1 antibody (magenta, middle image) in a *Lyve1-Cre mTmG* mouse. (B) Green fluorescent, *Lyve1-Cre*–positive cells associated with SC are macrophages, as indicated by their immunostaining with the macrophage marker IBA1. SC endothelium is immunolabeled with endomucin (magenta) and does not express GFP. (C) Higher magnification image showing the macrophage morphology of the GFP^+^ cells that are both next to and closely associated with SC. Scale bar, (A) 100 µm and (B–C) 50 µm.
(TIF)

Figure S7 Identification of SC in the *Wnt1-Cre mTmG* mouse. VECAD labeling along with size and location of the canal identify SC. The top image shows a red fluorescent structure (Figure 5), which immunolabels positively for VECAD (magenta) in the bottom image. VECAD shows distinct IW (tightly packed elongated thin cells) and OW (shorter wider cells) morphology, clearly identifying SC. Scale bar, 100 µm.
(TIF)

Figure S8 Organization of the LVP and RV at P1. Z-projections of confocal planes encompassing the LVP and RV show that the LVP has a complex architecture and the RVs are more linear. The LVP vessels run around the limbus largely in the same orientation as the future SC. The RVs run perpendicular to the LVP and are not restricted to the limbus but link up to the sinusoidal vessels of the developing ciliary body. The cornea is towards the bottom of the images. All images are from the same limbal segment of a *Kdr-GFP* mouse. Scale bar, 50 µm.
(TIF)

Figure S9 VEGFA localization during SC development. Matrix-bound VEGFA-164 localize as puncta to the developing SC at P2, an age of active tip cell formation and interaction. XY enface images are shown. Scale bar, 30 µm.
(TIF)

Figure S10 Macrophages and tip cells interact in the IZ during SC development. Macrophages are present at sites of tip cell interaction. A P2 whole mount was stained with endomucin (red) to mark tip cells and other endothelial cells and IBA1 (green) to label macrophages. An XY enface image is shown. The boxed regions are shown in more detail in Figure 8A. Scale bar, 50 µm.
(TIF)

Figure S11 Angiogenesis is active in the LVP when SC is starting its development. Tip cell interactions via their filopodia and the chaperoning of these interactions by macrophages are well established during angiogenesis. Macrophages are associated with sites of filopodial tip cell interactions in the LVP in this P2 eye. (Left) Panoramic Z-projection of LVP shows interacting tip cells emanating from capillaries stained with endomucin (red) and closely associated macrophages (IBA1^+^, green). (Right) Three examples of macrophages at sites of contact between tip cells (arrowheads). These regions correspond to the boxes in the merged image on the left. Scale bar, (Left) 50 µm and (Right) 20 µm.
(TIF)

Figure S12 Clusters of cells attached to the LV and RV colonize IZ. XY perspective of the P3 *Kdr-GFP* limbal segment shown in Figure 10. For completeness, the three indicated tissue depths are shown. The cornea is towards the bottom of the images. Cell clusters in the IZ are shown in the middle panels. Scale bar, 50 µm.
(TIF)

Figure S13 SC maturation and growth includes sprouting from the rSC. Z stacks encompassing rSC immunostained for endomucin show sprouts emerging from its sides at P5. The brightness was enhanced so that filopodia are readily visible. Three examples from different regions around an eye are shown. Sprouts (arrowheads) that emerge from the side of SC are rich in filopodia (thin arrows). Filopodia are also emerging from cells at the periphery of the developing SC that lack obvious sprouts (arrowheads, in A). The filopodia appear to mediate attachment between sprouts (block arrow in B) or between sprouts and the main body of the developing SC (block arrow in C). Based on nuclear staining, the sprouts are multicellular (not shown). Scale bar, 20 µm.
(TIF)

Figure S14 Limbal vessels are intact after sustained administration of KDR blocking antibody. Images are Z-projections of confocal stacks encompassing the LVP from the same limbal regions of the same eyes shown in Figure 17 (control antibody, IgG; function blocking antibody, DC101; two representative examples of each). The DC101- and control-injected eyes have a similar LVP. Note that there is substantial regional variation in LVP architecture in every eye. The RV was also unaffected. Thus, with this dose of DC101, the profound developmental impact of KDR inhibition on SC development is not secondary to a similar effect on the limbal vasculature. Scale bar, 100 µm.
(TIF)

## Abbreviations

ACAID: anterior chamber associated immune deviation
AQH: aqueous humor
BEC: blood endothelial cells
IOP: intraocular pressure
IZ: intermediate zone
LEC: lymphatic endothelial cells
LVP: limbal vascular plexus
PDPN: podoplanin
PROX1: prospero-related homeobox1
SC: Schlemm's canal
SEC: Schlemm's canal endothelial cells
VECAD: VE-cadherin
