# Supplementary figures and images for: Schlemm's Canal Is a Unique Vessel with a Combination of Blood Vascular and Lymphatic Phenotypes that Forms by a Novel Developmental Process
Source: PLoS Biol. 2014 Jul 22;12(7):e1001912. doi: 10.1371/journal.pbio.1001912 (PMC4106723; doi:10.1371/journal.pbio.1001912)

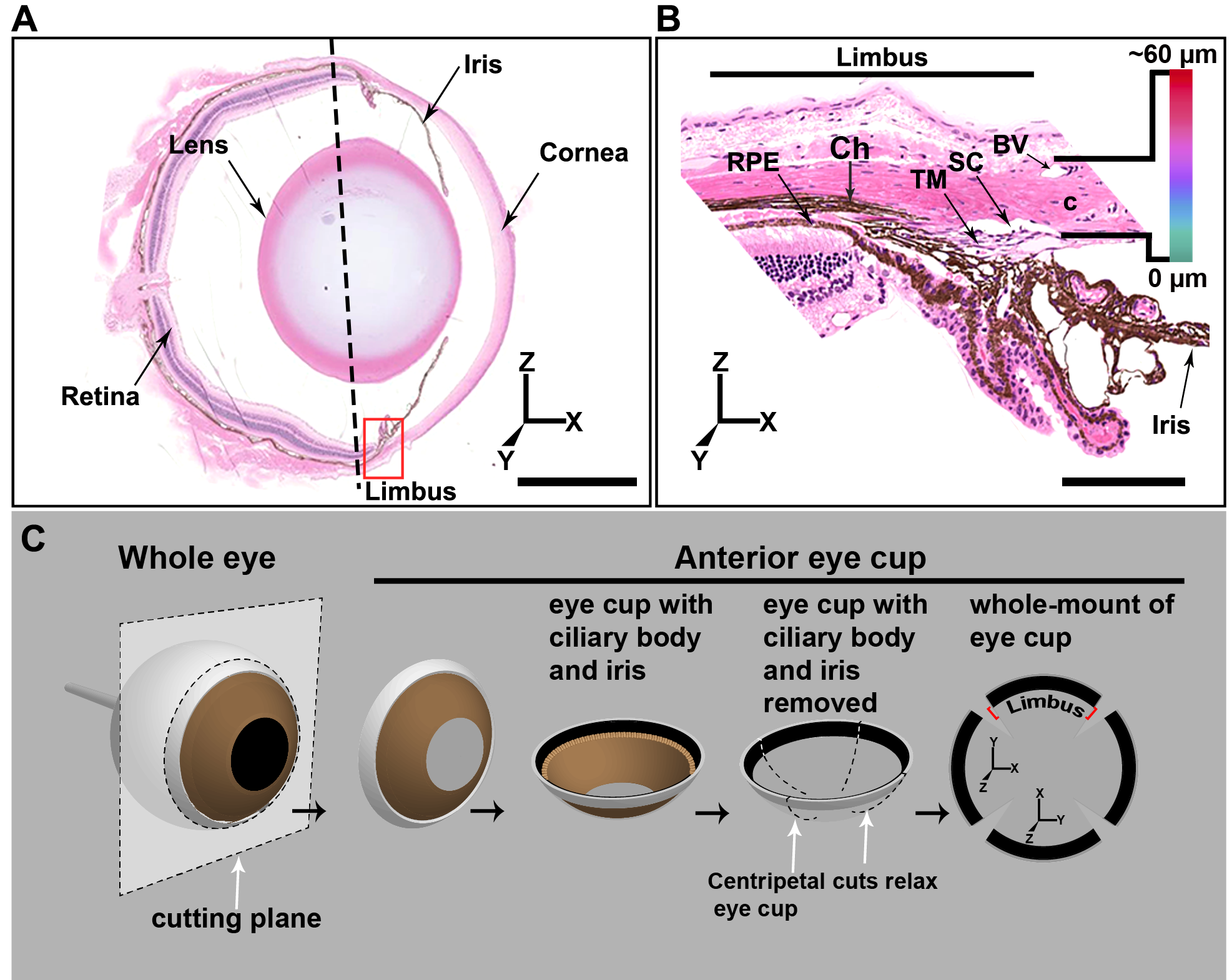

Supplement: Figure S1 — Orientation, depth coding, and whole mounting. (A) Sagittal section of the adult mouse eye stained with hematoxylin and eosin. The dotted line indicates the plane through which the front of the eye is separated from the posterior segment during dissection. XYZ coordinates are shown to provide orientation for all other figures throughout the article. (B) Higher magnification of the limbal region (red box in A), demonstrating the relative locations of trabecular meshwork (TM) and SC drainage structures in the iridocorneal angle. C, cornea; RPE, retinal pigmented epithelium; Ch, Choroid. The color scale indicates the relative color of depth-coded tissues in relation to the external surface of the eye in Figure 1 and all other figures where depth coding is used. Superimposing this scale on this conventional sectional view provides orientation for the location of SC and other structures in the 3D enface views elsewhere in the article. Note that redder colors represent tissues closer to the surface of the eye, whereas the cyan coded tissues are closer to the TM. SC is adjacent to the TM and due to mild local variation in its tissue depth color codes in blue to cyan. The blood vessels (BVs) that comprise the LVP are closer to the ocular surface than SC and depth code as magenta (see Figure 1B). (C) Whole mounting procedure. The eye is enucleated and the anterior eye cup dissected away along the cutting plane. Next, the lens, iris, and ciliary body are removed from the anterior eye cup. Centripetal cuts are made to relax the cup so that it lies flat. Red brackets indicate the location of the limbus and provide further orientation in relation to (B). The dark band around the periphery of the whole mount represents the pigment of the RPE, which remains due to the plane of separation of the cup from the rest of the eye. For all XYZ coordinates, the wedge indicates the dimension into and out of the plane of the paper, while the other coordinates are in the plane off the paper. [file pbio.1001912.s001.tif]

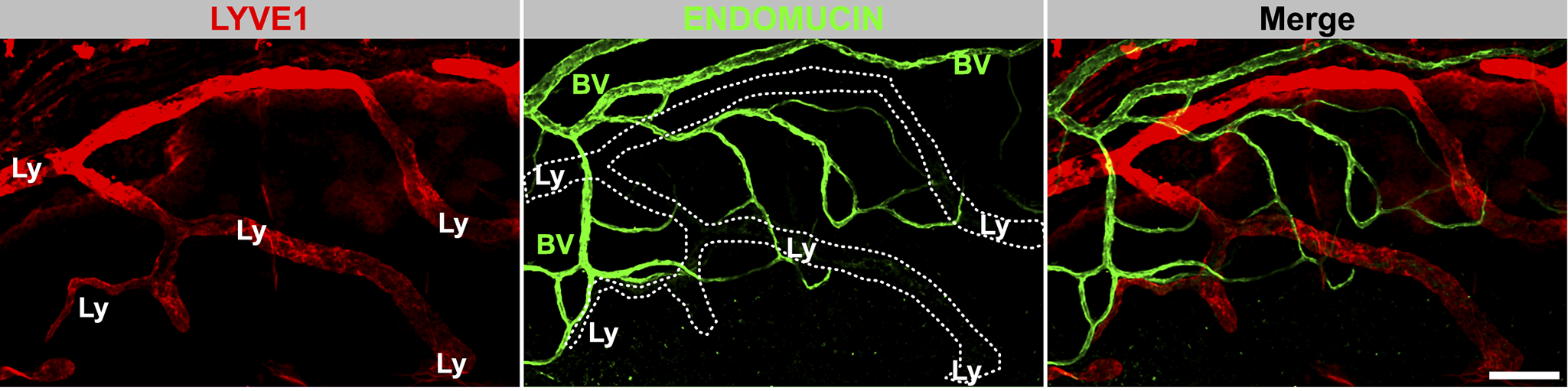

Supplement: Figure S2 — Endomucin is not expressed in lymphatic vessels. Z-projection of confocal planes encompassing the LVP and lymphatics show that endomucin (Middle) is robustly expressed in blood vessels but not lymphatics labeled with LYVE1 (Left). Position of the lymphatics is outlined in the middle image. BV, blood vessels; Ly, lymphatics. Scale bar, 100 µm. (TIF) [file pbio.1001912.s002.tif]

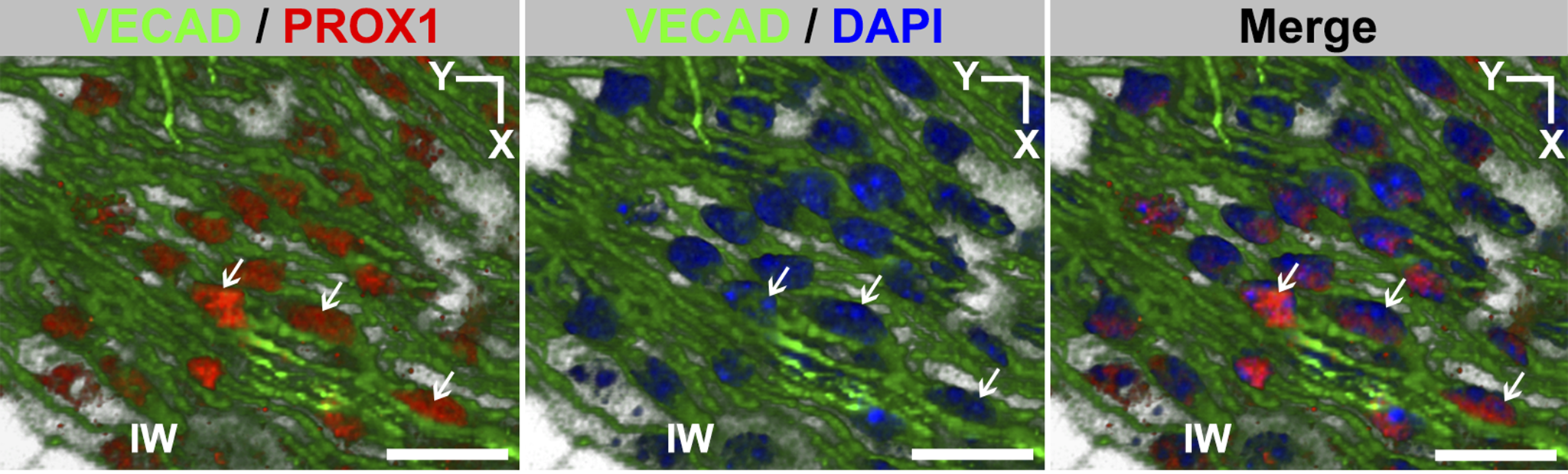

Supplement: Figure S3 — PROX1 expression in inner wall SCE. High-magnification confocal Z stacks of inner wall SCE rendered in 3D. Blend mode was used to give depth perception. The nuclei of VECAD-positive IW cells co-label for PROX1 (immunolabeling) and DAPI. As is well established, the DAPI-labeled nuclei bulge into the lumen of SC (towards the reader). Scale bars, 15 µm. (TIF) [file pbio.1001912.s003.tif]

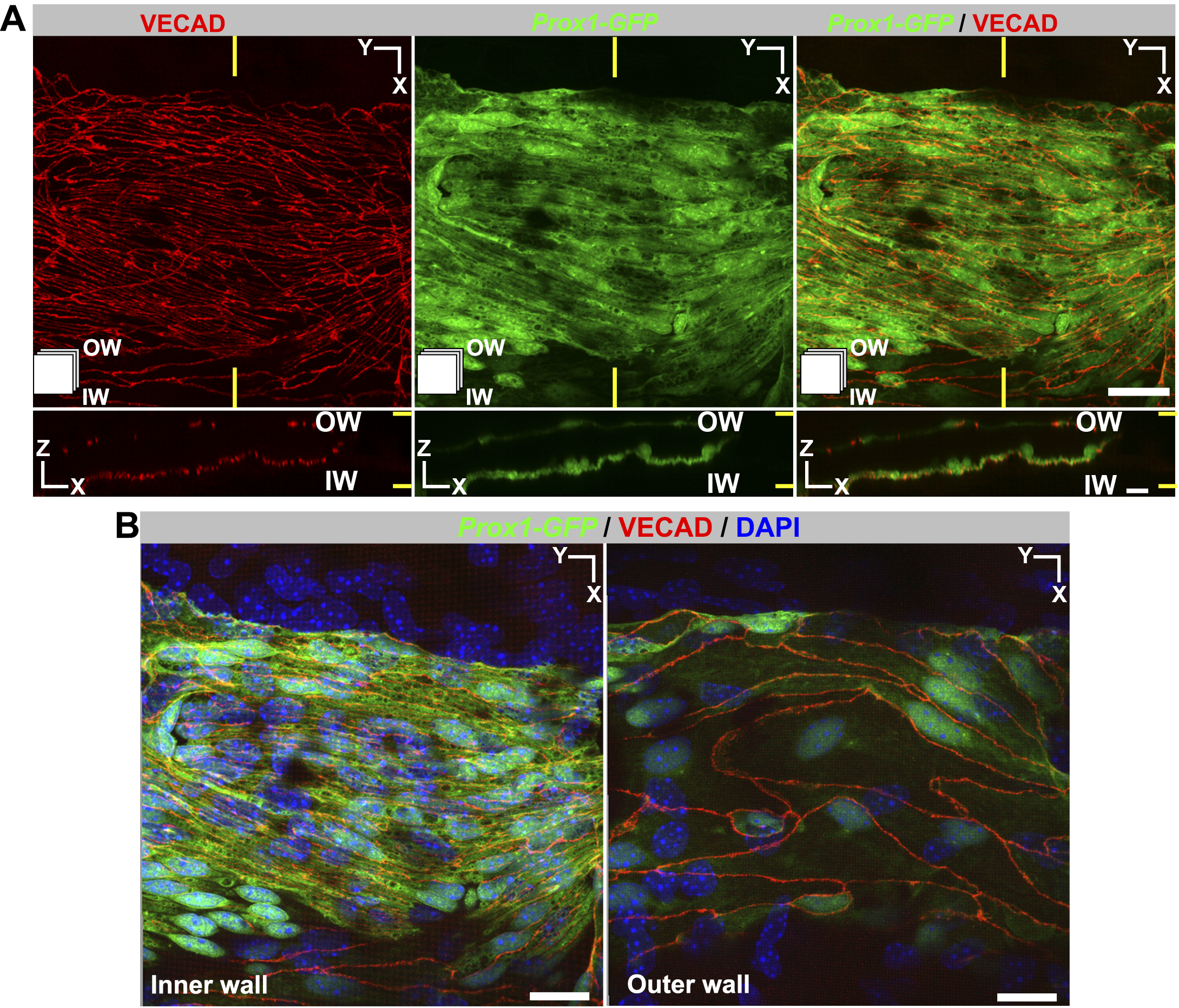

Supplement: Figure S4 — Primary localization of PROX1 in the inner wall of SC is confirmed using the Prox1-GFP transgenic mouse. (A and B) Prox1 expression is enriched in inner wall SCE. (A) High-magnification Z-projection of confocal planes encompassing SC in a Prox1-GFP mouse eye immunostained with VECAD. The projections show that the characteristically long and thin IW cells robustly express Prox1-GFP. XZ sections show enrichment of Prox1-GFP expression in the IW compared to OW (bottom images). The IW is easily distinguished by the close-packed VECAD puncta at cell junctions. In comparison, GFP fluorescence in the outer wall (well-spaced VECAD puncta) is weak. XZ sections are through the plane indicated by yellow lines in XY panels. (B) Z-projection of confocal planes encompassing either the IW (Left) or OW (Right) labeled with VECAD and DAPI confirm enrichment of Prox1-GFP expression in the thin IW cells versus wide OW cells. Note that the GFP label varies in its intracellular localization, sometimes being primarily in the nucleus and other times throughout the cell. Scale bar, (A–B) 20 µm. (TIF) [file pbio.1001912.s004.tif]

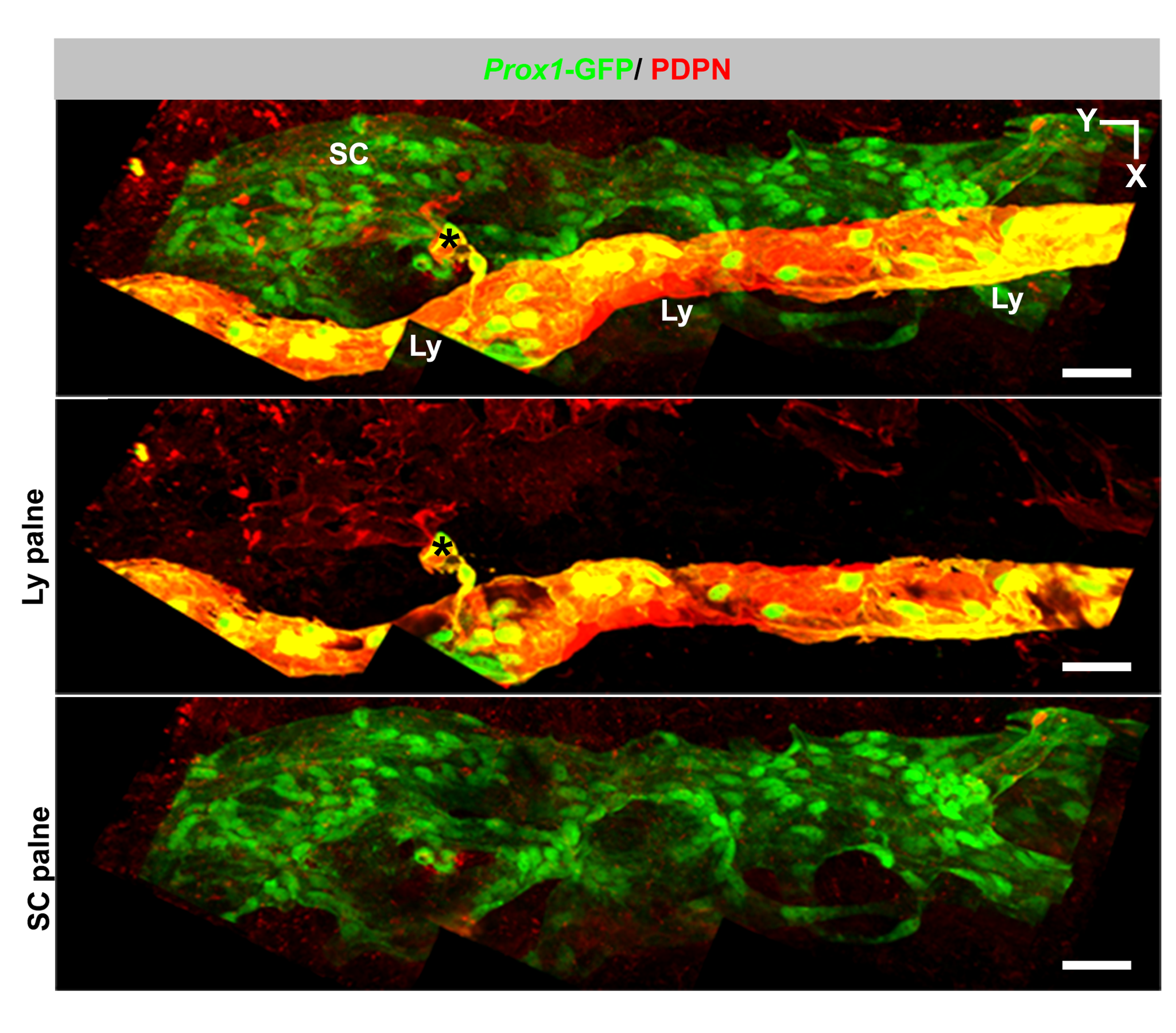

Supplement: Figure S5 — Podoplanin is not expressed in the SC. In an enface 3D view from the outer surface perspective of a Prox1-GFP eye, the lymphatics run on top of SC. In the top and middle images, co-labeling of podoplanin (PDPN) and Prox1-GFP renders the lymphatic vessel (Ly) a yellow color. *, lymphatic cells that have sheared off from the main vessel. SC is green as it expresses only Prox1-GFP but no podoplanin. Top image, 3D rendering showing the entire thickness of the limbal tissue has been rotated towards the viewer so that the lymphatics do not obscure SC. Middle image, Z-planes encompassing the lymphatic vessel. Bottom image, Z-planes encompassing the SC, Bottom image, podoplanin is not expressed in SC cells. Scale bar, 30 µm. (TIF) [file pbio.1001912.s005.tif]

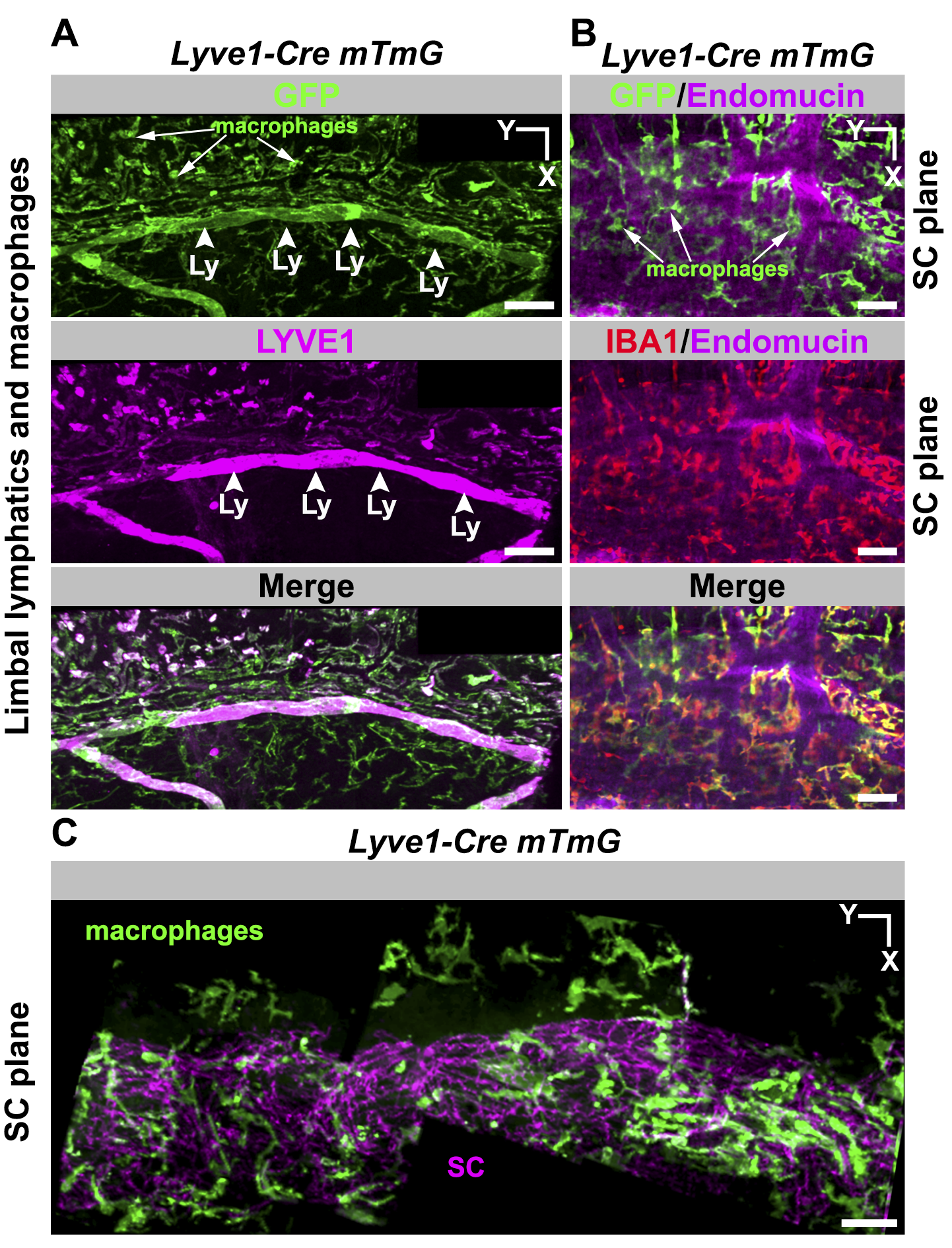

Supplement: Figure S6 — LYVE1 and IBA1 immunostaining confirm that the GFP+ cells associated with SC are macrophages. (A–B) Corresponding confocal planes at the levels of the indicated tissues from Lyve1-Cre mTmG mice. (A) Green fluorescent lymphatics (arrowheads, top image) and macrophages around them (arrows, top image) also immunostain with a LYVE1 antibody (magenta, middle image) in a Lyve1-Cre mTmG mouse. (B) Green fluorescent, Lyve1-Cre–positive cells associated with SC are macrophages, as indicated by their immunostaining with the macrophage marker IBA1. SC endothelium is immunolabeled with endomucin (magenta) and does not express GFP. (C) Higher magnification image showing the macrophage morphology of the GFP+ cells that are both next to and closely associated with SC. Scale bar, (A) 100 µm and (B–C) 50 µm. (TIF) [file pbio.1001912.s006.tif]

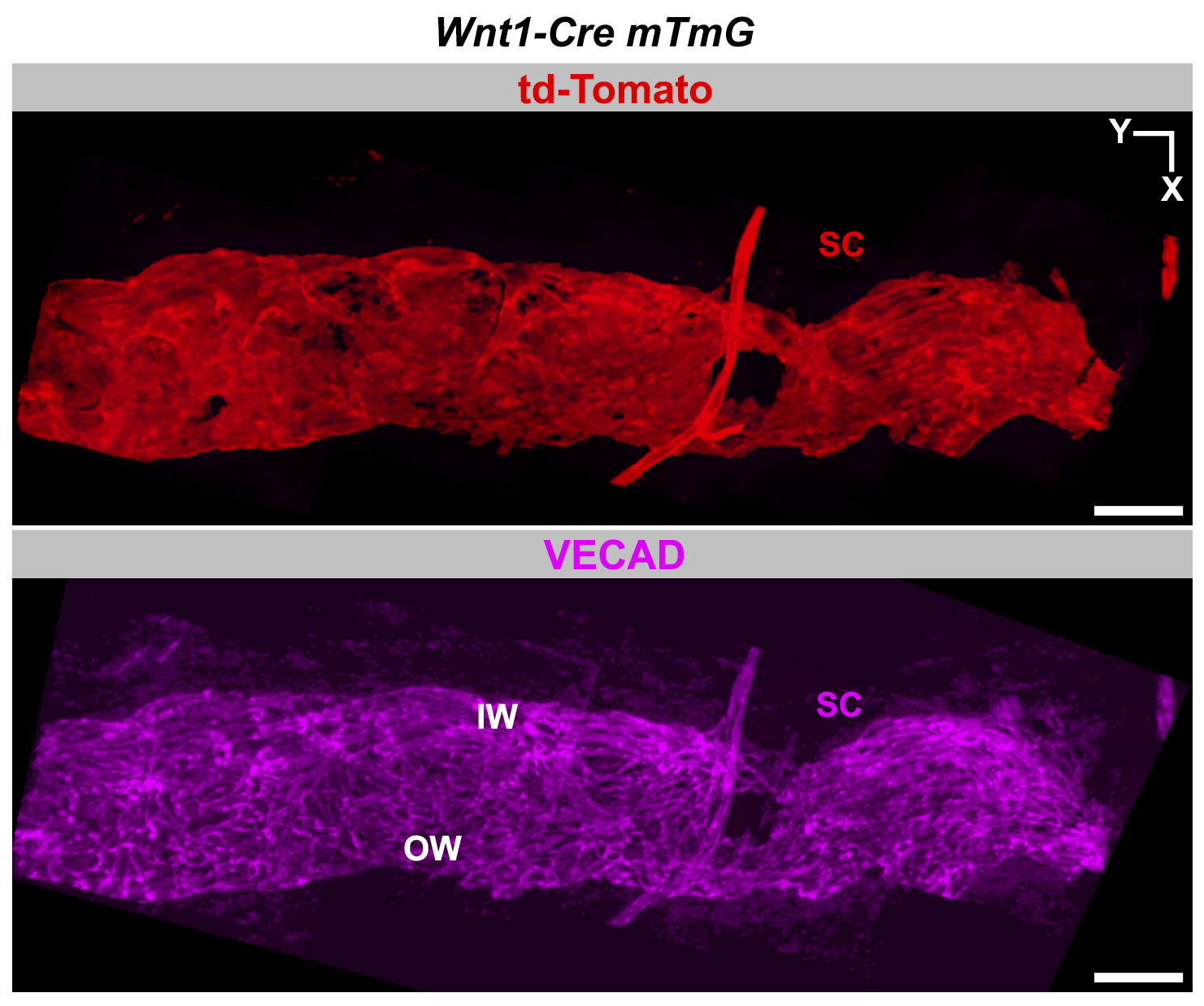

Supplement: Figure S7 — Identification of SC in the Wnt1-Cre mTmG mouse. VECAD labeling along with size and location of the canal identify SC. The top image shows a red fluorescent structure (Figure 5), which immunolabels positively for VECAD (magenta) in the bottom image. VECAD shows distinct IW (tightly packed elongated thin cells) and OW (shorter wider cells) morphology, clearly identifying SC. Scale bar, 100 µm. (TIF) [file pbio.1001912.s007.tif]

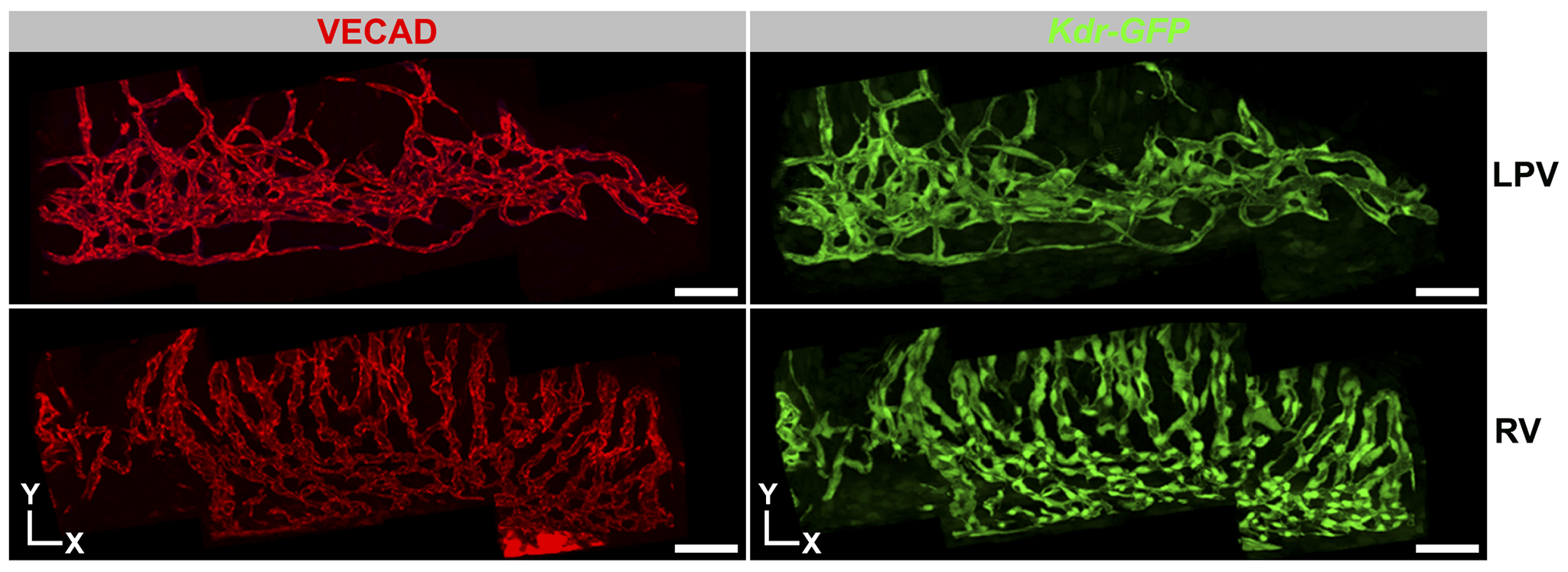

Supplement: Figure S8 — Organization of the LVP and RV at P1. Z-projections of confocal planes encompassing the LVP and RV show that the LVP has a complex architecture and the RVs are more linear. The LVP vessels run around the limbus largely in the same orientation as the future SC. The RVs run perpendicular to the LVP and are not restricted to the limbus but link up to the sinusoidal vessels of the developing ciliary body. The cornea is towards the bottom of the images. All images are from the same limbal segment of a Kdr-GFP mouse. Scale bar, 50 µm. (TIF) [file pbio.1001912.s008.tif]

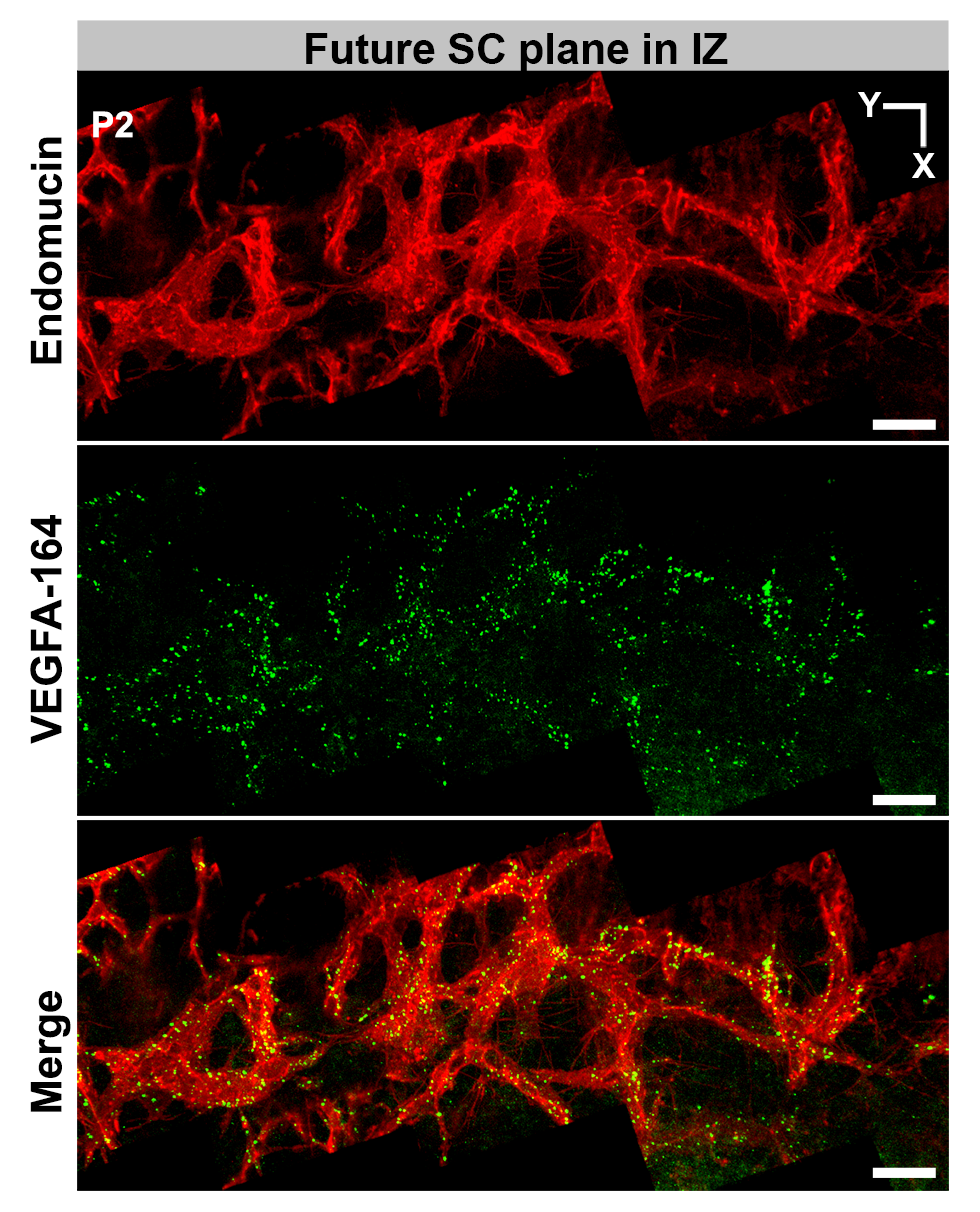

Supplement: Figure S9 — VEGFA localization during SC development. Matrix-bound VEGFA-164 localize as puncta to the developing SC at P2, an age of active tip cell formation and interaction. XY enface images are shown. Scale bar, 30 µm. (TIF) [file pbio.1001912.s009.tif]

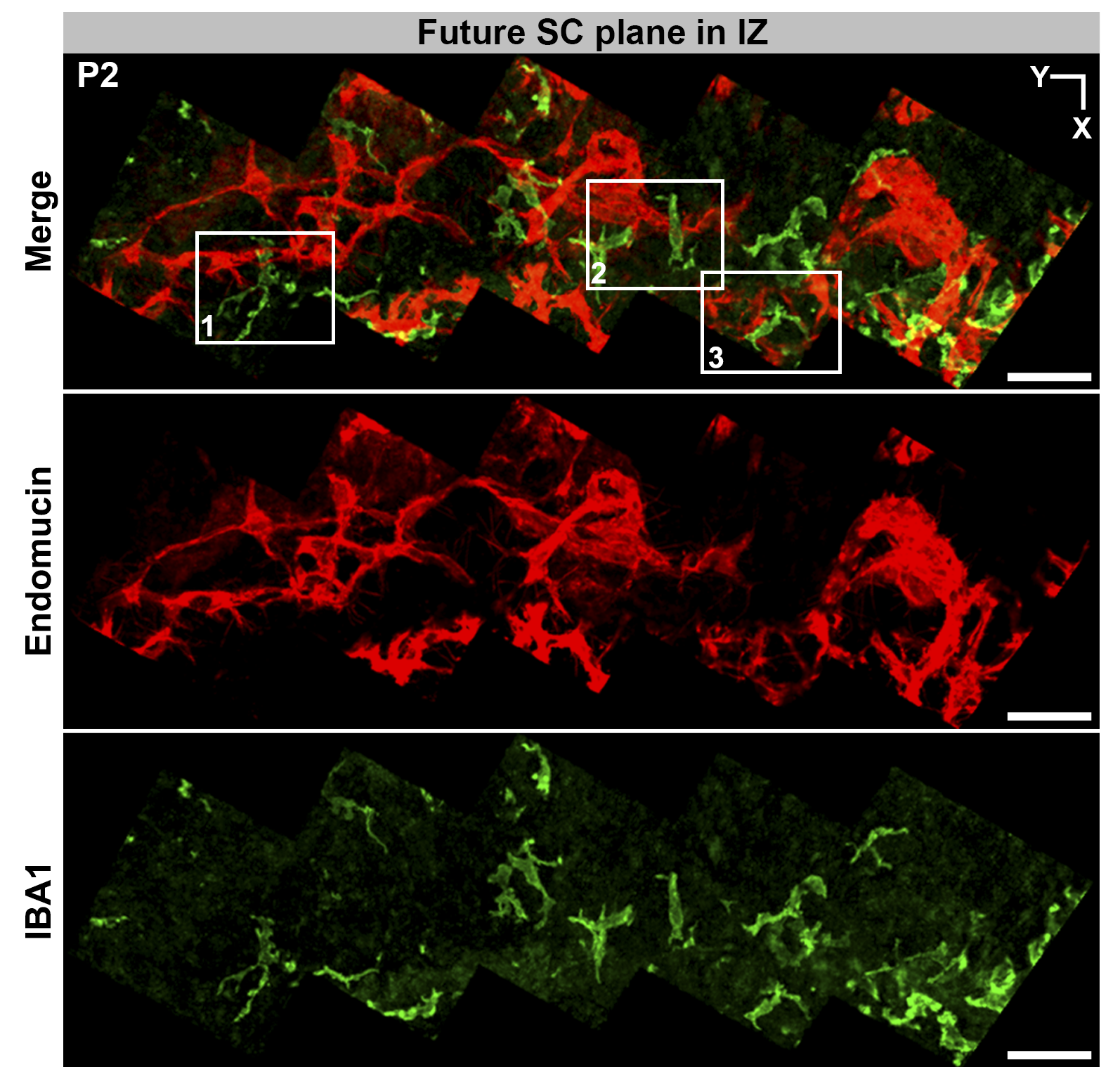

Supplement: Figure S10 — Macrophages and tip cells interact in the IZ during SC development. Macrophages are present at sites of tip cell interaction. A P2 whole mount was stained with endomucin (red) to mark tip cells and other endothelial cells and IBA1 (green) to label macrophages. An XY enface image is shown. The boxed regions are shown in more detail in Figure 8A. Scale bar, 50 µm. (TIF) [file pbio.1001912.s010.tif]

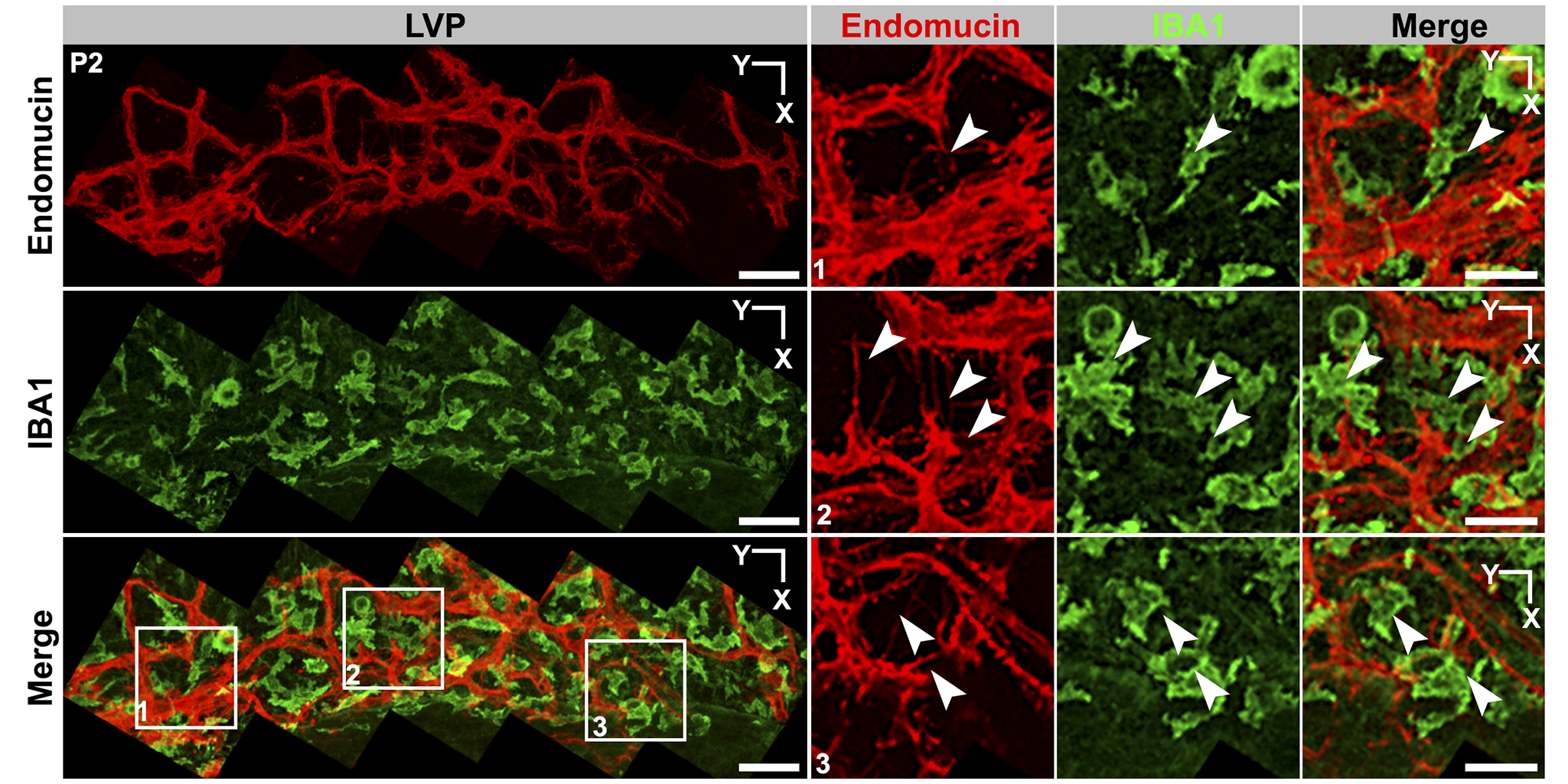

Supplement: Figure S11 — Angiogenesis is active in the LVP when SC is starting its development. Tip cell interactions via their filopodia and the chaperoning of these interactions by macrophages are well established during angiogenesis. Macrophages are associated with sites of filopodial tip cell interactions in the LVP in this P2 eye. (Left) Panoramic Z-projection of LVP shows interacting tip cells emanating from capillaries stained with endomucin (red) and closely associated macrophages (IBA1+, green). (Right) Three examples of macrophages at sites of contact between tip cells (arrowheads). These regions correspond to the boxes in the merged image on the left. Scale bar, (Left) 50 µm and (Right) 20 µm. (TIF) [file pbio.1001912.s011.tif]

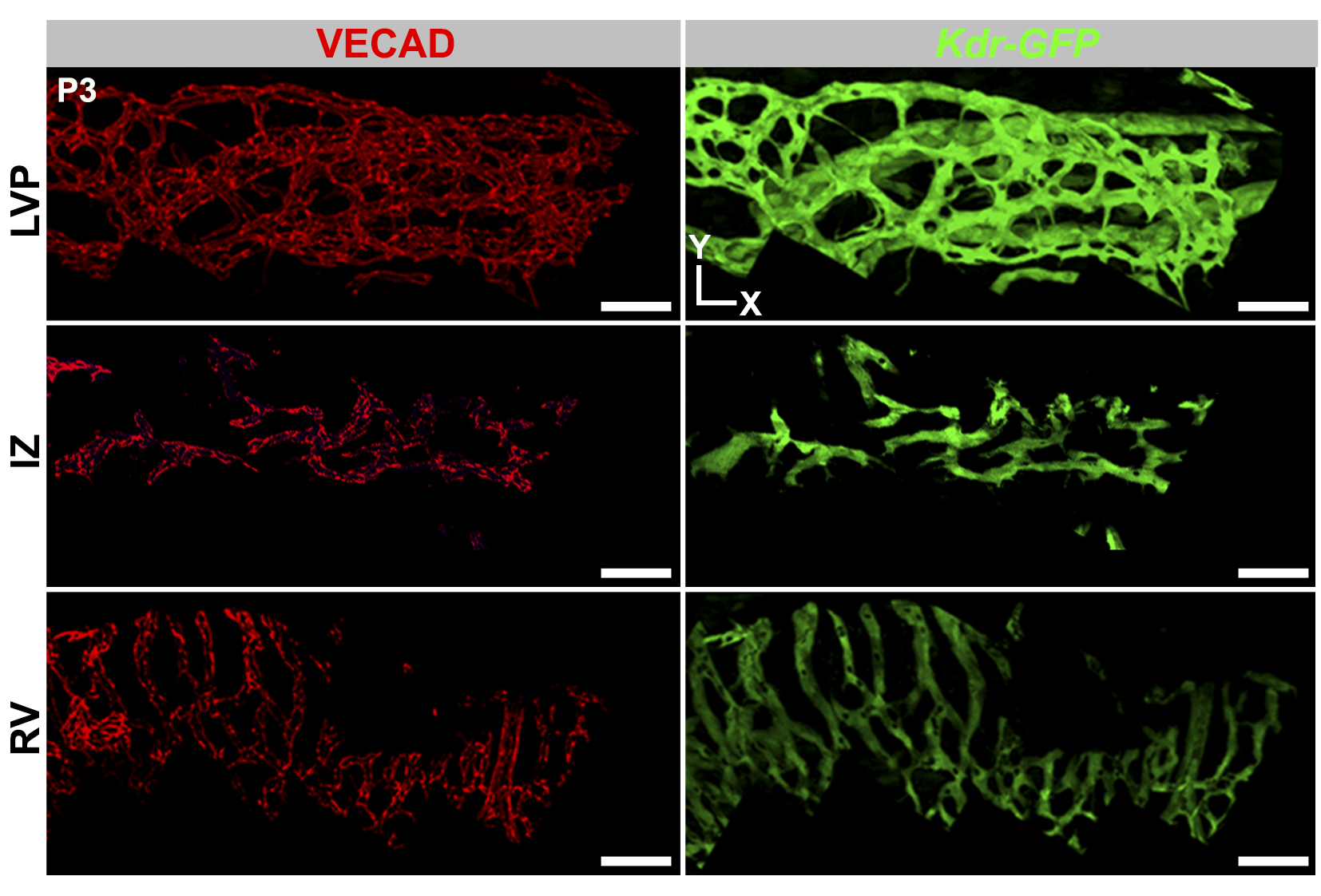

Supplement: Figure S12 — Clusters of cells attached to the LV and RV colonize IZ. XY perspective of the P3 Kdr-GFP limbal segment shown in Figure 10. For completeness, the three indicated tissue depths are shown. The cornea is towards the bottom of the images. Cell clusters in the IZ are shown in the middle panels. Scale bar, 50 µm. (TIF) [file pbio.1001912.s012.tif]

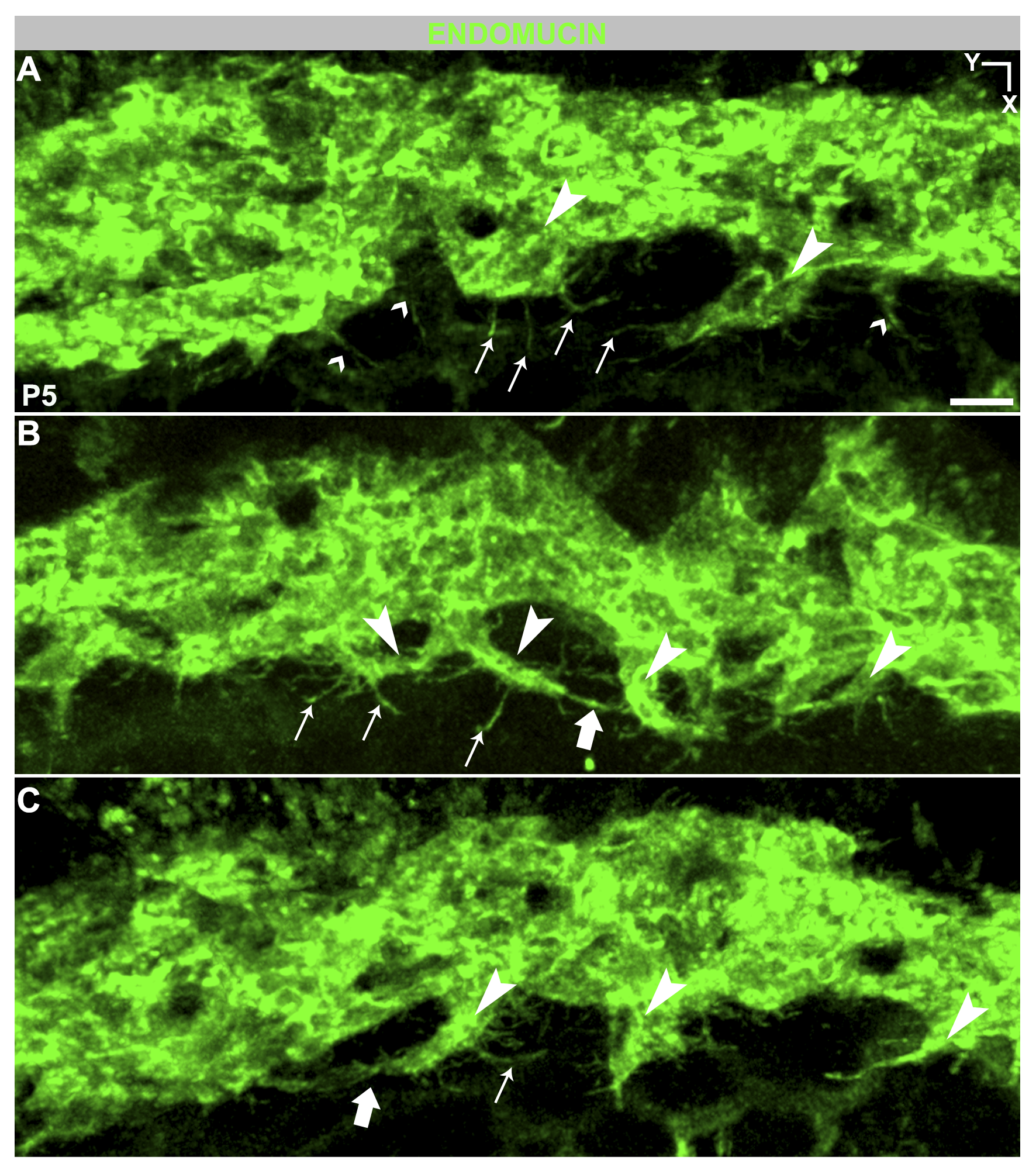

Supplement: Figure S13 — SC maturation and growth includes sprouting from the rSC. Z stacks encompassing rSC immunostained for endomucin show sprouts emerging from its sides at P5. The brightness was enhanced so that filopodia are readily visible. Three examples from different regions around an eye are shown. Sprouts (arrowheads) that emerge from the side of SC are rich in filopodia (thin arrows). Filopodia are also emerging from cells at the periphery of the developing SC that lack obvious sprouts (arrowheads, in A). The filopodia appear to mediate attachment between sprouts (block arrow in B) or between sprouts and the main body of the developing SC (block arrow in C). Based on nuclear staining, the sprouts are multicellular (not shown). Scale bar, 20 µm. (TIF) [file pbio.1001912.s013.tif]

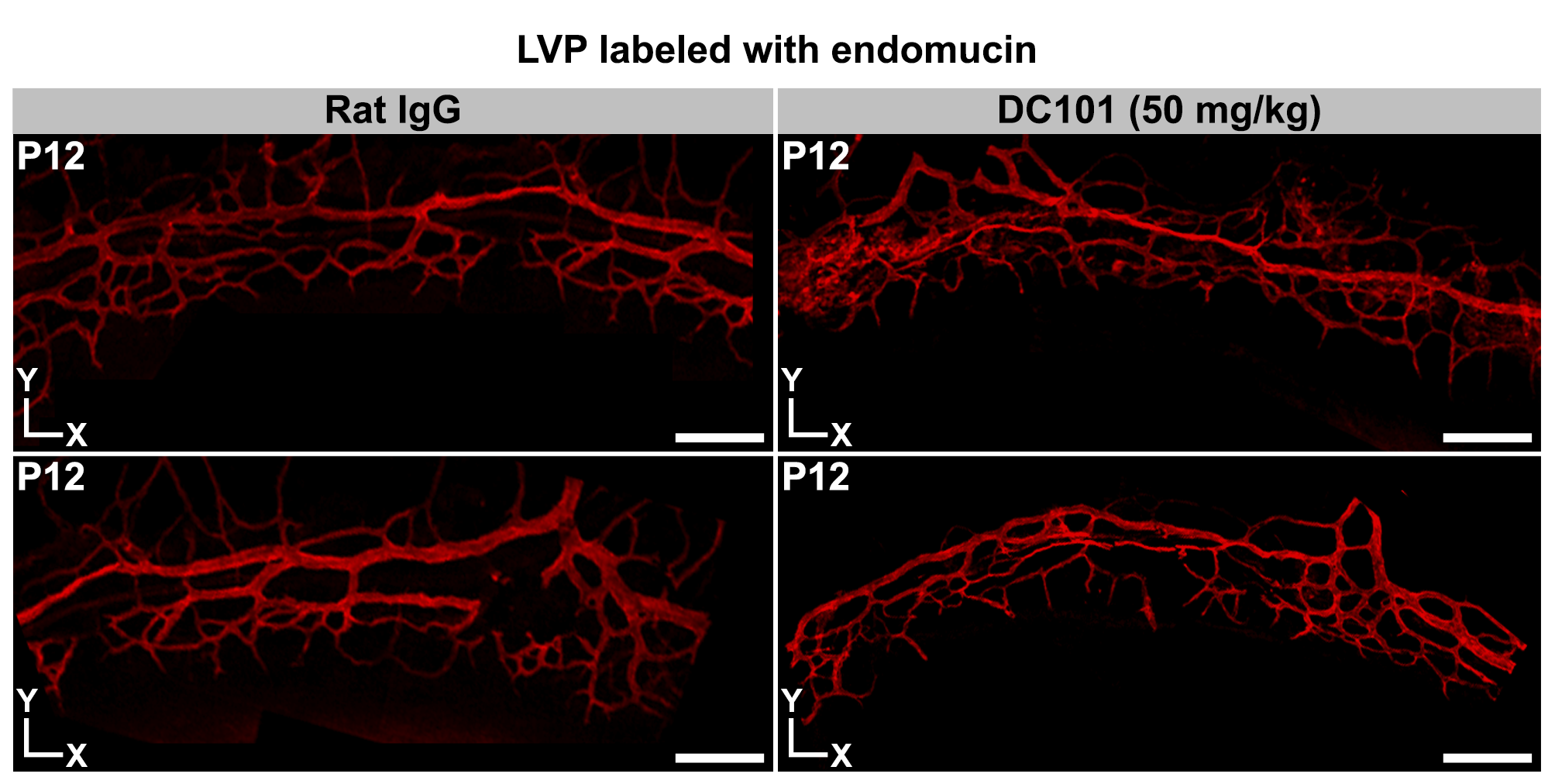

Supplement: Figure S14 — Limbal vessels are intact after sustained administration of KDR blocking antibody. Images are Z-projections of confocal stacks encompassing the LVP from the same limbal regions of the same eyes shown in Figure 17 (control antibody, IgG; function blocking antibody, DC101; two representative examples of each). The DC101- and control-injected eyes have a similar LVP. Note that there is substantial regional variation in LVP architecture in every eye. The RV was also unaffected. Thus, with this dose of DC101, the profound developmental impact of KDR inhibition on SC development is not secondary to a similar effect on the limbal vasculature. Scale bar, 100 µm. (TIF) [file pbio.1001912.s014.tif]
